# Phage Cocktail in Combination with Kasugamycin as a Potential Treatment for Fire Blight Caused by *Erwinia amylovora*

**DOI:** 10.3390/antibiotics11111566

**Published:** 2022-11-06

**Authors:** Sang-Guen Kim, Sung-Bin Lee, Su-Jin Jo, Kevin Cho, Jung-Kum Park, Jun Kwon, Sib Sankar Giri, Sang-Wha Kim, Jeong-Woo Kang, Won-Joon Jung, Young-Min Lee, Eunjung Roh, Se-Chang Park

**Affiliations:** 1 Laboratory of Aquatic Biomedicine, Research Institute for Veterinary Science, College of Veterinary Medicine, Seoul National University, Seoul 08826, Korea; 2 Crop Protection Division, National Institute of Agriculture Sciences, Rural Development Administration, Wanju 55365, Korea

**Keywords:** phage–antibiotic synergy, bacteriophage, phage therapy, *Erwinia amylovora*, fire blight

## Abstract

Recently, there has been an increasing number of blight disease reports associated with *Erwinia amylovora* and *Erwinia pyrifoliae* in South Korea. Current management protocols that have been conducted with antibiotics have faced resistance problems and the outbreak has not decreased. Because of this concern, the present study aimed to provide an alternative method to control the invasive fire blight outbreak in the nation using bacteriophages (phages) in combination with an antibiotic agent (kasugamycin). Among 54 phage isolates, we selected five phages, pEa_SNUABM_27, 31, 32, 47, and 48, based on their bacteriolytic efficacy. Although only phage pEa_SNUABM_27 showed host specificity for *E. amylovora*, all five phages presented complementary lytic potential that improved the host infectivity coverage of each phage All the phages in the cocktail solution could lyse phage-resistant strains. These strains had a decreased tolerance to the antibiotic kasugamycin, and a synergistic effect of phages and antibiotics was demonstrated both in vitro and on immature wound-infected apples. It is noteworthy that the antibacterial effect of the phage cocktail or phage cocktail-sub-minimal inhibitory concentration (MIC) of kasugamycin was significantly higher than the kasugamycin at the MIC. The selected phages were experimentally stable under environmental factors such as thermal or pH stress. Genomic analysis revealed these are novel *Erwinia*-infecting phages, and did not encode antibiotic-, virulence-, or lysogenic phage-related genes. In conclusion, we suggest the potential of the phage cocktail and kasugamycin combination as an effective strategy that would minimize the use of antibiotics, which are being excessively used in order to control fire blight pathogens.

## 1. Introduction

The bacterium *Erwinia amylovora* is a causative agent of fire blight, a devastating disease of rosaceous plants [1,2]. Fire blight-free regions suffer devastating economic losses following the first outbreak of fire blight invasion due to there being no specific methods to effectively control plant pathogens, except for a limited number of antibiotics such as streptomycin, oxytetracycline, and kasugamycin [3,4,5]. *E. amylovora* isolates from apple orchards are known to have resistance to streptomycin, the primary treatment for fire blight [6]. Furthermore, the high prevalence of resistance genes to these antibiotics in the environment (endosphere, rhizosphere, or phyllosphere), creates a high probability of the transfer of antibiotic resistance genes to pathogens [7,8,9]. Consequently, a high concentration of antibiotics should be used to be effective against bacterial outbreaks, including fire blight, which can cause dysbiosis in the environmental microbiota. A disrupted microbial balance can facilitate an outbreak of diseases [10,11].

To overcome the problem of antibiotic resistance in *E. amylovora*, a number of alternatives have been reported, such as essential oil, plant extracts, and antagonistic bacteria [12,13,14,15,16,17]. In addition, bacteriophages (phages) have been suggested as potential alternatives to antibiotics for controlling fire blight owing to their direct killing effect [18,19]. The comparative advantages of using phages to control pathogens mainly comprise their ability to specifically recognize cell surface receptors on their bacterial hosts to infect and lyse the pathogen after replication within the host cell [20,21]. For decades, a number of phages have been characterized as effective agents against fire blight, and several commercial phages have been developed and made available worldwide as solutions against fire blight such as Omnilyticus AgriPhage™-Fire Blight (Salt Lake City, UT, USA) and Enviroinvest Erwiphage PLUS (Kertváros, Hungary) [22,23,24]. The high specificity of therapeutic phages confers on them the advantage of being able to be used as a biocontrol method without affecting beneficial microbes in the environment. However, it can be a major limitation at the same time owing to the inability of such phages to act on a broad range of pathogens. Therefore, phages with a broad host range are generally preferred for therapeutic use [25,26,27].

Combining different phages in cocktail solutions is the primary strategy to overcome the limitation of the narrow host range of phage therapy [28,29]. Different phages in cocktail solutions can complement the host range coverage of every other phage in the solution, as well as address the issue of phage resistance being developed due to the administration of a single type of phage [30,31]. In particular, phage cocktails are expected to show a synergistic effect of the combination of phages, although this is not always observed to be the case [32,33]. Another strategy is to combine antibiotics with phages [34]. Synergy between phages and antibiotics can be demonstrated to occur by the observation of enhanced plaque size or clarity, and improved growth characteristics of phages, such as a shortened eclipse period or increased burst size [35,36,37,38,39]. The application of phages to control bacterial pathogens can therefore reduce the excessive use of antibiotics, thus, allowing them to be reserved for urgent clinical needs.

The present study investigated the biocontrol potential of newly isolated *Erwinia* phages. With the five phages selected in our study, we showed the effectiveness of the resultant phage cocktail, as well as that of its combination with antibiotics, which we propose as an alternative strategy to control fire blight caused by *E. amylovora*.

## 2. Results

### 2.1. Bacteriophage Screening

Among 54 phage isolates, we selected five phages that showed the highest growth-inhibitory effect against *E. amylovora* TS3128, which is the reference strain for fire blight research in South Korea. A low concentration 5 log colony forming unit [CFU]/mL of *E. amylovora* was co-cultured with phages for the first screening. Phages pEa_SNUABM_2, 6, 14, 15, 16, 18, 20, 23, 26, 27, 28, 30, 31, 32, 36, 38, 40, 42, 43, 44, 46, 47, 48, 49, 50, 52, and 54 were selected as cocktail candidates in the initial screening. Subsequently, a high concentration (6 log CFU/mL) of *E. amylovora* was used for the second screening. The five phages pEa_SNUABM_27 (vB_EaM-SNUABM_27; φ27), pEa_SNUABM_31 (vB_EaM-SNUABM_31; φ31), pEa_SNUABM_32 (vB_EaM-SNUABM_32; φ32), pEa_SNUABM_47 (vB_EaM-SNUABM_47; φ47), and pEa_SNUABM_48 (vB_EaM-SNUABM_48; φ48) were selected as the final cocktail candidates in the second screening round (Supplementary Table S1).

### 2.2. Morphological and Biological Characteristics of the Bacteriophages

**Figure 1:**
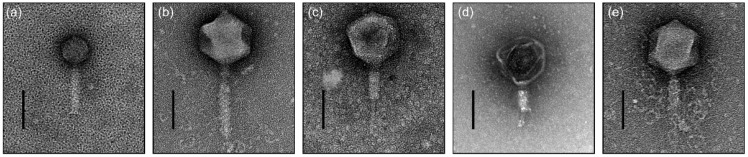
Transmission electron micrographs of *Erwinia* bacteriophages (**a**) φ27, (**b**) φ31, (**c**) φ32, (**d**) φ47, and (**e**) φ48. Scale bar is 100 nm. The contractile tails of φ32, φ47, and φ48 were observed in the contracted state (**c**–**e**).

The selected phages were morphologically recognized as belonging to the family *Myoviridae* (Figure 1). Extended long tail fibers were observed around φ48 (Figure 1e). Structural observations of phages φ27, φ31, φ32, φ47, and φ48, showed the presence of a capsid having diameter minimum 68.5 ± 2.76 nm and maximum 139.15 ± 5.47 nm, and a contractile tail having length minimum 115.1 ± 2.16 nm and maximum 196.32 ± 11.45 nm (*n* = 5) (Table 1). The host range of the five selected phages is represented in using 94 and 25 isolates of *E. amylovora* and *E. pyrifoliae*, respectively. All five phages infected 100% of the *E. amylovora* strains (94/94) recently isolated in South Korea. Although φ27 showed a narrow host range when tested against *E. pyrifoliae* strains (8/25; 32%), other phages could complement the host coverage, rendering all *E. pyrifoliae* strains susceptible to the infectivity of those phages (Supplementary Materials Figure S1).

### 2.3. In Vitro Bacterial Killing Assay

**Figure 2:**
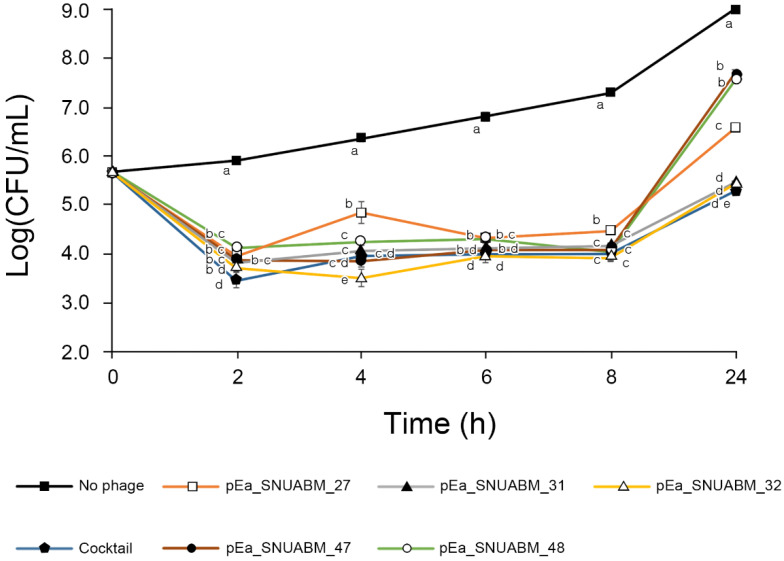
In vitro bactericidal effect of *Erwinia* phages φ27, φ31, φ32, φ47, φ48, and their cocktail. The viable bacterial cells were counted over 24 h. The *E. amylovora* strain TS3128, a reference strain for research in Korea, was used. The bars of each point indicate the standard deviation. Statistical significance was calculated using the one-way analysis of variance test with Tukey post-hoc, and the significance threshold was set at *p* < 0.05. Means at the same sampling time point with different letters (a–e) are significantly different.

Phage administration led to a rapid lysis of *E. amylovora* (Figure 2). Each phage was effective in lysing *E. amylovora* up to 8 h; however, the regrowth of *E. amylovora* was observed at 24 h of incubation with φ27, φ47, and φ48. The CFU of regrown bacteria in samples treated with the cocktail, φ31, and φ32 was significantly lower than that of the samples treated φ27, φ47, and φ48 (*p* < 0.05). The phage cocktail contained 1/5 parts concentration of each phage, and yet was an extremely effective solution for inhibiting the pathogen. The administration of the single phages resulted in 2.4, 3.5, 3.5, 1.2, 1.4 log CFU/mL reduction in the final bacterial counts of phages φ27, 31, 32, 47, and 48, respectively, and the bactericidal effect of the five-phage cocktail led to a 3.7 log CFU/mL reduction of the bacterial count, which is a significant decrease compared with the bacterial count of the untreated control group (*p* < 0.001 at 2 h, 4 h, 6 h, 8 h, and 24 h).

### 2.4. Biological Characteristics of Phage-Resistant Erwinia amylovora TS3128 Derivatives

**Figure 3:**
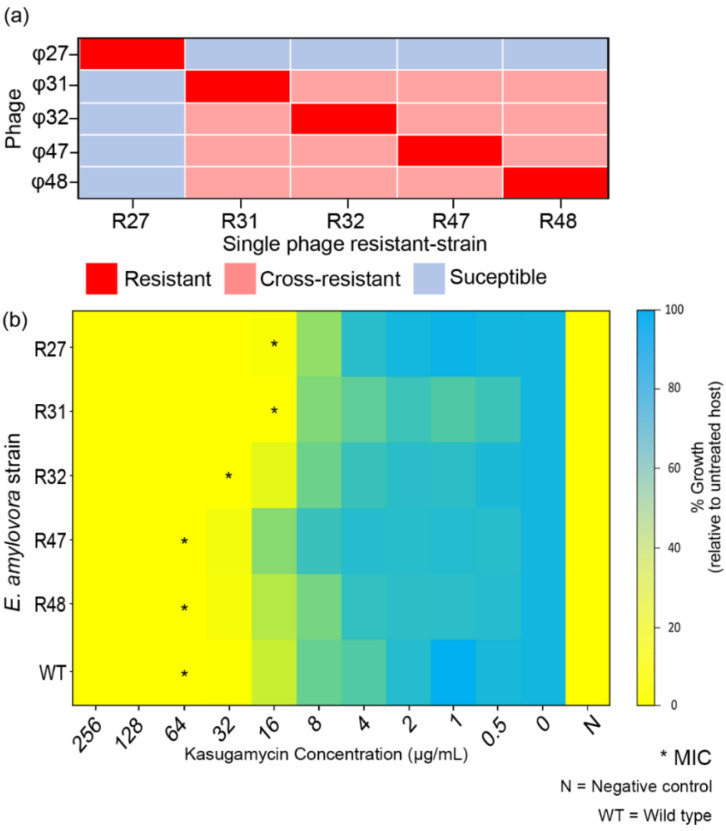
Biological characteristics of phage-resistant *Erwinia amylovora* TS3128. (**a**) Phage resistance profiles of the single phage-resistant strains. (**b**) Minimum inhibitory concentration (MIC) of kasugamycin with the phage-resistant strains is indicated (*). WT and N indicate wild type and negative control (no bacterial ingredient), respectively.

The profile of phage susceptibility of single phage-resistant strains is summarized in Figure 3a. The R27 strain was susceptible to phages φ31, 32, 47, and 48. While the phage resistance of R31, R32, R47, and R48 was induced by φ31, 32, 47, and 48, respectively, the resistant strains gained cross-resistance to all the other unrelated phages (φ31, 32, 47, and 48) except φ27 (Figure 3a). However, the cocktail solution infected all phage-resistant strains.

The minimal inhibitory concentration (MIC) values of kasugamycin against the wild type *E. amylovora* TS3128 and the phage-resistant strains are shown in Figure 3b. The MIC of kasugamycin for wild type (WT) TS3128 was observed to be 64 μg/mL. Moreover, we observed a 2- to 4-fold decrease in the MIC of the phage-resistant strains R27, R31, and R32.

### 2.5. Phage–Antibiotic Synergy (PAS) Assay

**Figure 4:**
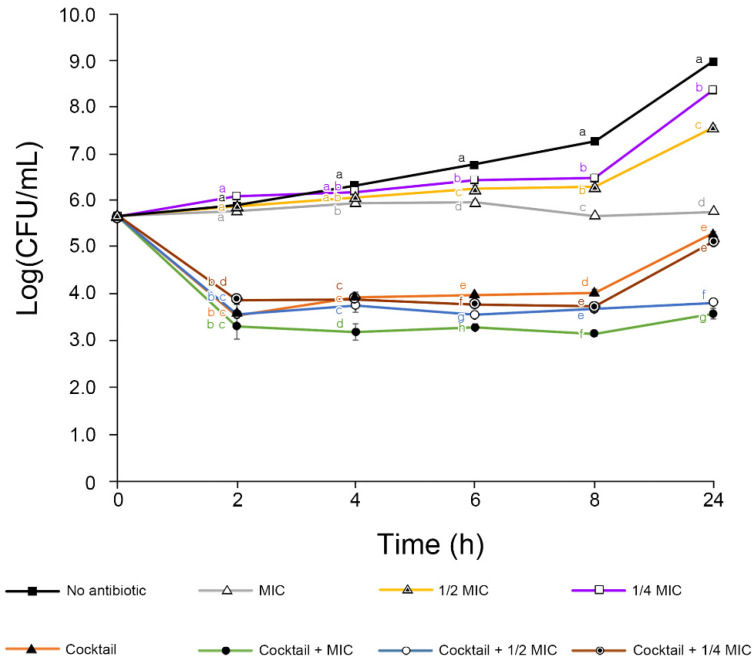
In vitro phage cocktail–antibiotic synergy assay with *Erwinia amylovora* TS3128. The viable bacterial cells were counted over 24 h. The bars of each point indicate the standard deviation. Statistical significance was calculated using the one-way analysis of variance test with Tukey post-hoc, and the significance threshold was set at *p* < 0.05. Means at the same sampling time point with different letters (a–g) are significantly different.

To determine the enhanced antibacterial activity of the phage cocktail with kasugamycin, a phage–antibiotic synergy assay was performed with different antibiotic concentrations and the bacteria TS3128 (Figure 4). Kasugamycin at MIC inhibited the growth of *E. amylovora*, while sub-MIC inoculations allowed bacterial growth. There was a slight enhancement in the antibacterial effect from using the phage cocktail and 1/4 MIC kasugamycin combination. The advanced effect was much higher when the phage cocktail was combined with 1/2 MIC and 1 MIC kasugamycin. The final viable bacterial cell count reduction was 3.7 (phage cocktail), 3.8 (phage cocktail–1/4 MIC kasugamycin), 5.1 (phage cocktail–1/2 MIC kasugamycin), and 5.4 (phage cocktail–1 MIC kasugamycin), therefore resulting in the PAS effect (difference of bacterial cell count reduction between phage cocktail only and phage cocktail–kasugamycin combination) to be 0.1 (phage cocktail–1/4 MIC kasugamycin), 1.4 (phage cocktail–1/2 MIC kasugamycin), and 1.7 (phage cocktail–1 MIC kasugamycin). The samples treated using the phage cocktail with 1/4 MIC, 1/2 MIC, and 1 MIC kasugamycin showed a significant reduction compared with treated with kasugamycin alone (*p* < 0.001).

### 2.6. Experiment on Apple Fruit under Controlled Conditions

**Figure 5:**
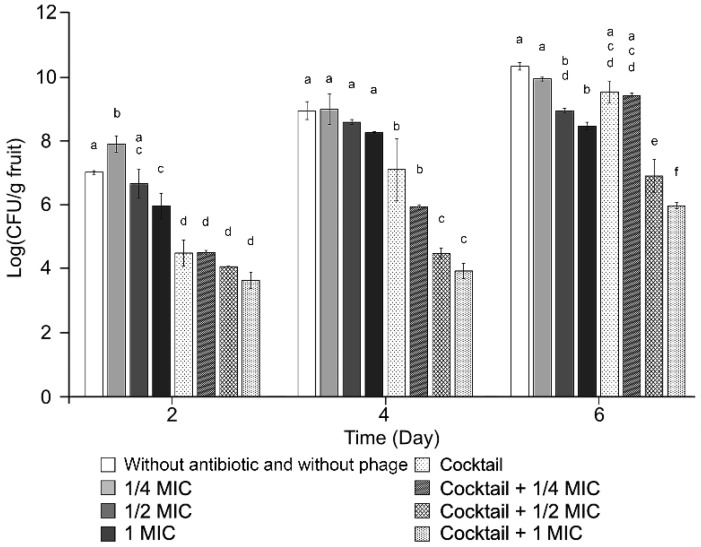
Apple fruit administration of the five-phage cocktail in combination with 0, 1/4, 1/2, or 1 MIC kasugamycin, under controlled conditions. The infective concentration of *Erwinia amylovora* TS3128 was 2 × 10^5^ Colony Forming Unit [CFU]/mL. Viable bacterial cell counts were observed over time. The bars of each point indicate the standard deviation. Statistical significance was calculated using the one-way analysis of variance test with Tukey post-hoc, and the significance threshold was set at *p* < 0.05. Means at the same sampling time point with different letters (a–f) are significantly different.

A biocontrol assay of the phages was conducted for the phage cocktail and its combination with antibiotics (Figure 5). A significant improvement in inhibition of bacterial growth was observed when the phage cocktail–kasugamycin combination with 1/2 MIC and 1 MIC was administered compared with the phage cocktail treatment at day 4 and 6 (*p* < 0.001). The final viable bacterial cell count reductions were 0.39 (1/4 MIC kasugamycin), 1.37 (1/2 MIC kasugamycin), 1.86 (1 MIC kasugamycin), 0.7 (phage cocktail), 3.4 (phage cocktail–1/2 MIC kasugamycin), and 4.35 (phage cocktail–1 MIC kasugamycin).

### 2.7. Stability Assay

The stability of the phages under environmental stressors (pH and temperature) was examined. A majority of the five phages were considerably stable under the thermal conditions tested (4–50 °C), except for phages φ32 and φ48, which are both slightly vulnerable to high temperatures (50 °C; Figure S2). In addition, the infectivity of φ32 was slightly hindered under alkaline conditions, while the other phages were stable under different pH conditions ranging from pH 4 to 9 (Figure S2).

### 2.8. Genomic Analysis of the Selected Phages

The general features of the genomes of the phages are presented in Table 1. The five *Erwinia* phages φ27, φ31, φ32, φ47, and φ48 possessed double-stranded circular DNA, having a guanine–cytosine (GC) content of 44.07%, 49.53%, 49.19%, 34.48%, and 49.52%, respectively. Phage φ27 possessed a relatively small genome (53,014 bp) compared with the those of other phages, and the *Erwinia* jumbo phage φ47 had a large genome (355,376 bp). In total, 78, 337, 336, 540, and 358 open reading frames (ORFs) were identified in the genomes of φ27, 31, 32, 47, and 48, respectively. The genus of φ27 was identified as *Loessnervirus*, characterized by a genome of 55.80 kbp with 44.2% GC content, such as the *Erwinia* phage vB_EamM-Y2 and the *Pantoea* phage vB_PagM_SSEM1. No encoded tRNAs have been previously reported in the genomes of *Loessnervirus*; however, one tRNA was identified in the genome of φ27. The genus of φ31 and φ32 was identified as *Alexandravirus*, represented by the *Erwinia* phage Alexandra and the *Dickeya* phage AD1. This genus presents genomes of 261–266 kbp coding two distinct tail sheath proteins. Phages φ31 and φ32 have two tail sheath proteins and no tRNA. The genome of φ47 was identified as *Eneladusvirus*, represented by the *Serratia* phage BF and the *Yersinia* phage Yen9-04. This genus presents a genome of 354–357 kb with 34.4% GC contents and 35 tRNAs. Phage φ48 has two tRNAs but its genome was unclassified.

**Figure 6:**
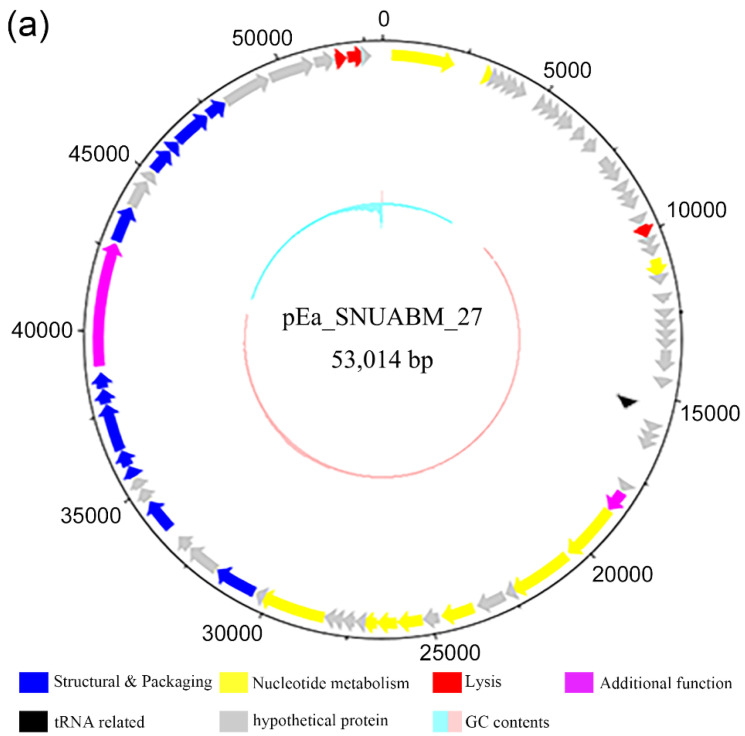

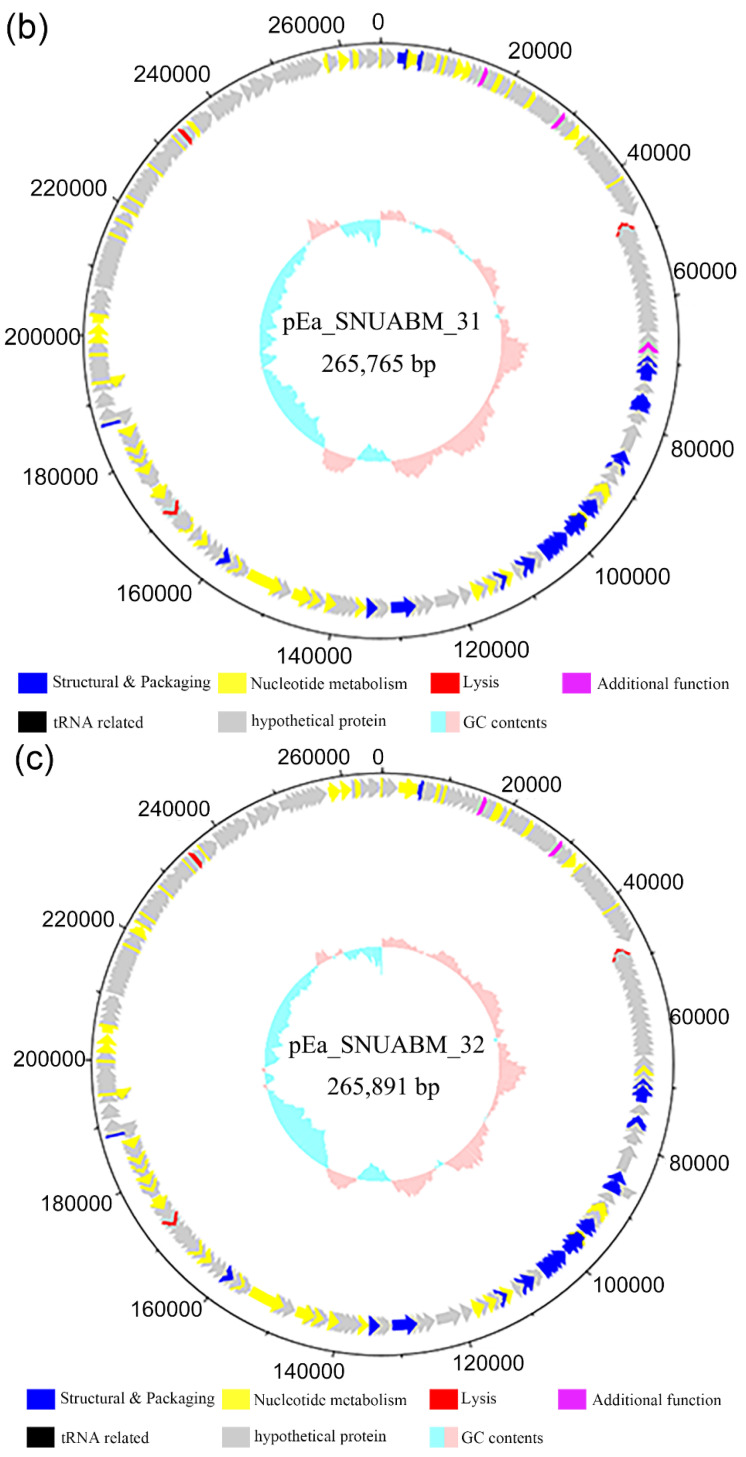

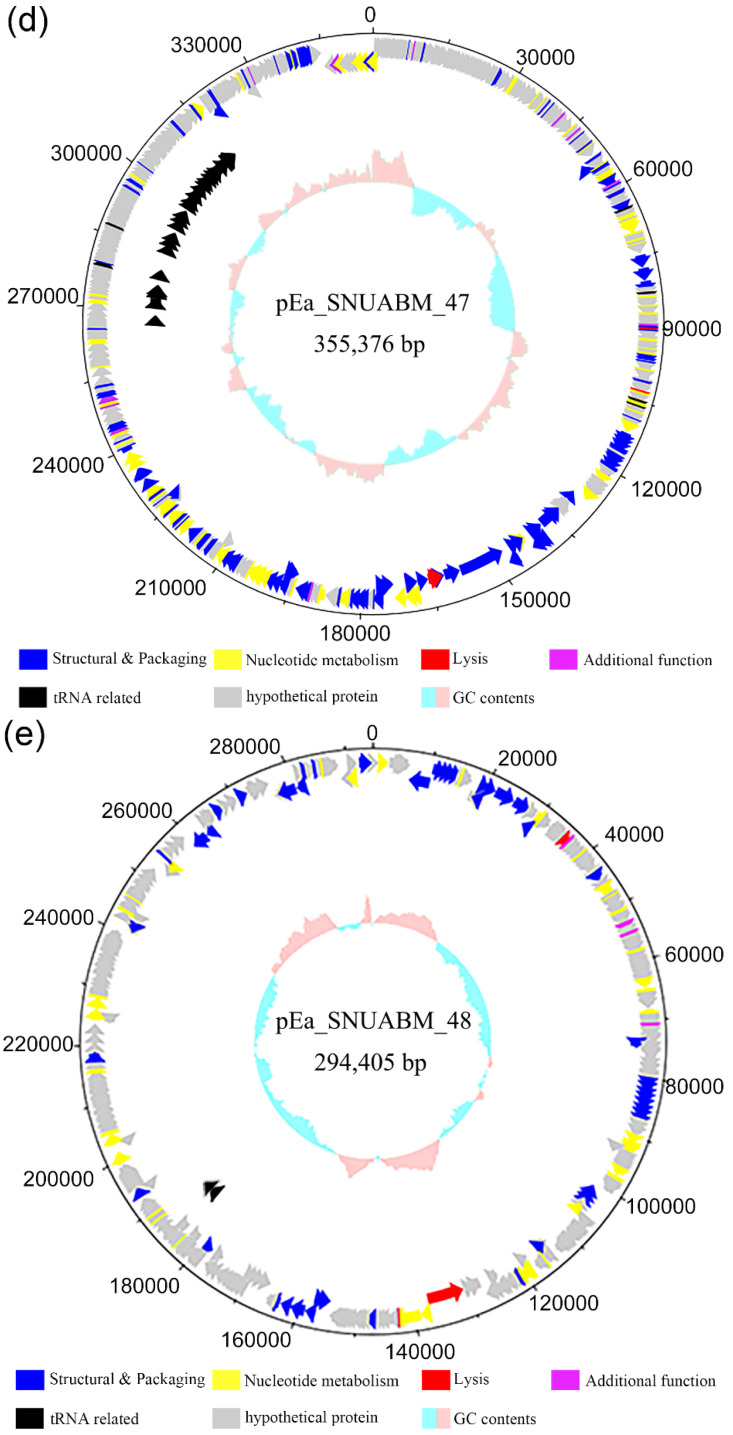
Genome map of *Erwinia* phages (**a**) φ27, (**b**) φ31, (**c**) φ32, (**d**) φ47, and (**e**) φ48. The open reading frames were functionally assorted into six groups of proteins related to: structure and packaging (blue), nucleotide metabolism (yellow), lysis (red), and additional functions (purple), as well as tRNA proteins or tRNA-related proteins (black), and hypothetical proteins (gray). Scale is base pair (bp).

The *Erwinia* phages in this study showed dissimilar and unique genomic arrangements, except for phages φ31 and φ32, as they were in the same genus. Even though most of the predicted ORFs had no matches in any database, identified proteins from the five phages could be categorized into the following six groups based on their functions: proteins related to structure and packaging, nucleotide metabolism, tRNA, lysis, additional functions, and hypothetical proteins (Figure 6, Tables S2–S6).

### 2.9. Comparative Genomic Analysis

**Figure 7:**
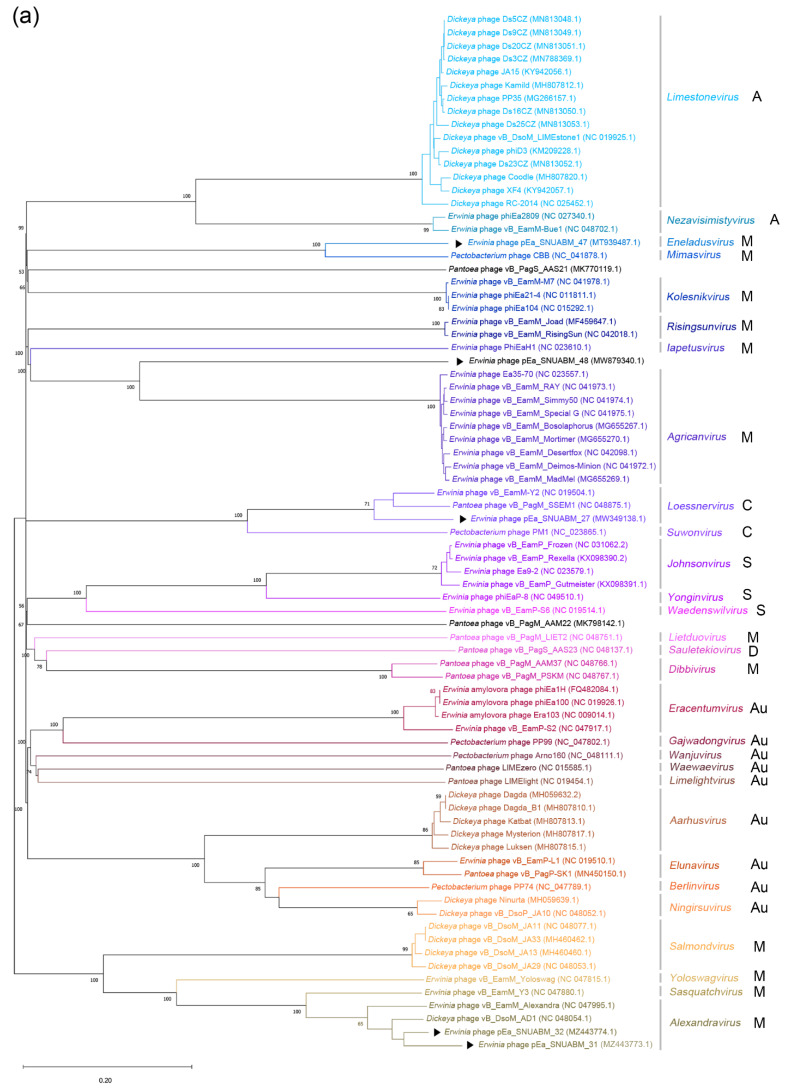

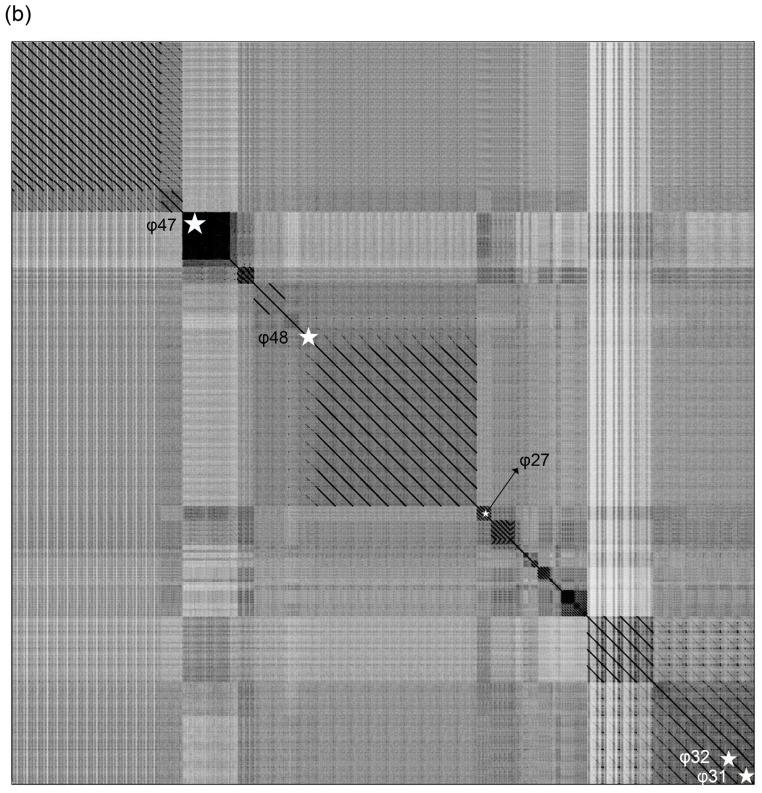

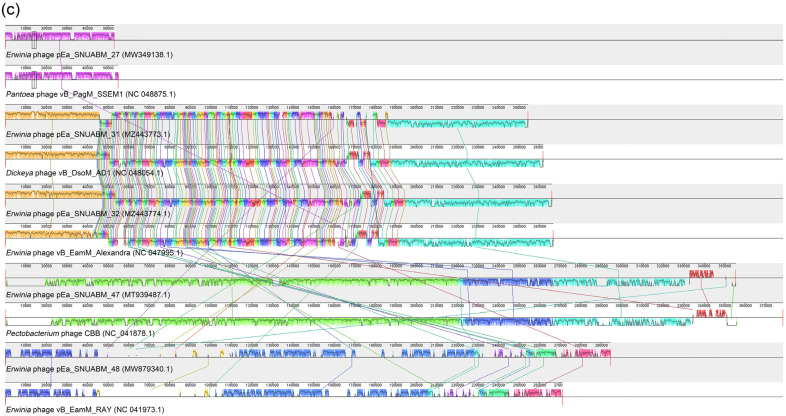
Phylogenetic whole genome analysis of 79 phages infecting *Erwinia* spp., *Dickeya* spp., *Pantoea* spp., and *Pectobacterium* spp. (**a**) The phylogenetic tree was constructed using Virus Classification and Tree Building Online Resource (VICTOR). Black arrows (▶) indicate the five *Erwinia* phages in this study. Black letters next to genus indicate family of the phages (A: *Ackermannviridae*, M: *Myoviridae*, C: *Chaseviridae*, S: *Schitoviridae*, D: *Drexlerviridae*, Au: *Autographiviridae*). The genus of φ27 was identified as *Loessnervirus*, that of φ31 and φ32 as *Alexandravirus*, that of φ47 as *Eneladusvirus*, while that of φ48 was unclassified. (**b**) Dot plot analysis of the 79 phages with parallel order of phylogeny. (**c**) Comparative whole genome analysis using progressive Mauve.

The whole-genome sequences of the five phages were evaluated for comparative analysis with representative phages infecting *Erwinia* spp., *Dickeya* spp., *Pantoea* spp., and *Pectobacterium* spp. A phylogenetic analysis using the Virus Classification and Tree Building Online Resource (VICTOR) clustered the phages according to their taxonomy (Figure 7a). Phage φ27 was clustered with *Erwinia* phage vB_EamM-Y2 (NC 019504.1) and *Pantoea* phage vB_PagM_SSEM1 (NC 048875.1), in a manner similar to the clustering exhibited by *Loessnervirus*. The cluster comprising φ31 and φ32 was clustered with *Dickeya* phage vB_DsoM_AD1 (NC 048054.1), and these two phages were identified as *Alexandravirus*. Phage φ47 was clustered with *Pectobacterium* phage CBB (NC_041878.1) and identified as *Eneladusvirus*. Phage φ48 formed a distinct cluster that diverged from a common ancestor with *Agricanvirus* bacteriophages. The dot plot analysis of the 79 phages indicated firm clustering and supported the phylogenetic analysis (Figure 7b). Phage φ27 had a strong lineage association with *Loessnervirus* (*Erwinia* phage vB_EamM-Y2 and *Pantoea* phage vB_PagM_SSEM1); phages φ31 and φ32 were seen to be closely related to *Alexandravirus* (*Erwinia* phage vB_EamM_Alexandra and *Dickeya* phage vB_DsoM_AD1). In contrast, phages φ47 and φ48 did not demonstrate close relatedness with other reported *Erwinia* phages.

Progressive Mauve was used to align and compare phages φ27, φ31, φ32, φ47, and φ48 with genetically close phages: *Pantoea* phage vB_PagM_SSEM1, *Dickeya* phage vB_DsoM_AD1, *Erwinia* phage vB_EamM_Alexandra, *Pectobacterium* phage CBB, and *Erwinia* phage vB_EamM_RAY (Figure 7c). The genome of φ27 and *Pantoea* phage vB_PagM_SSEM1 were identified as the *Loessnervirus* genus. The genomes of φ31, *Dickeya* phage vB_DsoM_AD1, φ32, and *Erwinia* phage vB_EamM_Alexandra were closely related with the genus *Alexandravirus*. Furthermore, close relatedness of φ47 with *Pectobacterium* phage CBB was also determined. A comparative study between the genomes of φ48 and *Erwinia* phage vB_EamM_RAY (*Agricanvirus*) was conducted, since the genus of φ48 was not identified in the genomic analysis; this showed similarity with, however, relevant differences. The results showed that the genome sequences of φ27, φ31, φ32, φ47, and φ48 presented the differences from their closest relatives, which supported the comparative results from the phylogenetic analysis and dot plot analysis.

## 3. Discussion

Fire blight was first reported in 2015, and since then there has been an increasing number of outbreaks in South Korea, especially recently [40,41]. Without any regulations regarding the administration order for antibiotics to control the fire blight in South Korea, secondary agents, including kasugamycin, are widely used in general. There are no investigations that reported the antibiotic resistance of *E. amylovora* in South Korea, however, misuse of the antibiotic agents can promote the evolution of resistance, and dysbiosis of the orchard environment, which would lead to the failure of the fire blight management of the nation. To combat this severe blight disease, our research team has been dedicated to developing phages as effective alternatives to antibiotics. Due to the presence of two nearly indistinguishable pathogens, *E. amylovora* and *E. pyrifoliae*, in South Korea, phages that are capable of infecting both pathogens are considered ideal biocontrol agents.

Although the phages used in this study were isolated using *E. amylovora* as their host, they could infect *E. pyrifoliae*, an endemic species that also led to blight symptoms in plants in South Korea, which is in accordance with the previous reports that *Erwinia amylovora* bacteriophages have a broad host range [22,23,42,43,44,45,46]. From our *Erwinia* phage isolates, we screened phages based on their bacterial cell lysis efficacy and selected phages φ27, φ31, φ32, φ47, and φ48 to form the *Erwinia* phage cocktail solution. Phages in the cocktail improved each other’s host range complementarily, leading the cocktail to be infective towards all recently isolated *E. amylovora* and *E. pyrifoliae* strains. Combining phages with complementary host ranges is one of the key virtues of phage cocktails, since phages present host-specific infectivity [28].

The ideal strategy for phage cocktails is to generate synergy between phages [28]. As the phages inhibit the secondary infection (superinfection) of their close relatives, it is a crucial factor to exclude the ones revealing the antagonistic effect in the cocktail [28]. One promising way to generate synergism is combining the phages having virion-associated enzymes [32]. In line with the prediction that the genome of pEa_SNUABM_47, a constituent of cocktail, encodes for tail spike lysozyme [45]. Indeed, pEa_SNUABM_47 revealed the synergistic effect in the first phases (0 to 8 h) of the in vitro bacterial killing assay with the phages that are genetically distant (Figure 2 and Figure 6). Even though this effect could not be achieved over the long term (24 h), the selected phages did not show an antagonistic effect, which is not recommended for cocktail constituents [28].

Analysis of phage resistance in the five phages showed cross-resistance between φ31, φ32, φ47, and φ48 (Figure 3). Only the φ27-resistant strain (R27) did not show cross-resistance with other phages, and vice versa. It is remarkable that phages selected from distinct genera could be cross-resistant (Figure 7a). The genomic arrangement of the five phages was totally unrelated; eventheir lysis-related proteins did not show homology to each other (Figure 7b,c, and Tables S2–S6). The infection process of the phages was considered to be the origin of the phage resistance and the cross-resistance. However, this contradicts previous presumptions of infection mechanisms differing based on the taxonomical status (family) of the phages [45]. As of today, a number of phages have been reported, and genomic classifications have been improved and updated. In our study, all phages were classified in the family *Myoviridae*; however, the host recognition strategy of the myophages with a small genome is presumed to differ from that of jumbo myophages. More detailed analyses are warranted in future studies to elucidate host–phage interactions. We suggest that analyzing cross-resistance patterns among candidate phages for cocktail solutions should be considered as the highest priority. Because the phages’ host preference and infection have a dependency on exopolysaccharides (EPS) produced by *E. amylovora*, a novel strategy combining the strains that produce different amounts of EPS have been suggested for the host range analysis [46,47].

Even though resistance to antimicrobial agents is a major concern, the phages in our cocktail solution could control the phage-resistant strains (Figure 3a). Bacterial pathogens might acquire phage resistance by fitness trade-off [48]. To escape contact with phages, bacteria modify (or even lose) receptors used for phage infection as their first-line anti-phage defense strategy [49]. Often, these alterations cause lowered viability, decreased pathogenicity, and increased susceptibility to antimicrobial agents [50]. Interestingly, a trade-off between phage resistance and kasugamycin susceptibility was observed in the phage-resistant *E. amylovora* strains R27, R31, and R32. The decreased MIC is indicative of PAS against *E. amylovora*. Indeed, the phage–antibiotic combination proved to have superior efficacy in both the in vitro and apple fruit assays, which may reduce the use of antibiotics in the field. PAS was observed even at sub-inhibitory antibiotic concentrations (Figure 4 and Figure 5).

Aminoglycoside antibiotics, such as gentamicin, kanamycin, streptomycin, and kasugamycin, are translation-interfering drugs that can also hinder translation in phages, resulting in premature lysis [51,52]. Even worse from the perspective of phage therapy, in the long term aminoglycosides can cause the extinction of phages from the environment [53]. However, the antibiotic action of kasugamycin is competitive [54], therefore translation can be initiated if surplus initiation factors are present. Such translational initiators include initiation tRNA (tRNAi), such as tRNA-fMET, which is encoded in the jumbo *Erwinia* phage pEa_SNUABM_47 [45]. The synergy and facilitation between phages and kasugamycin is presumed to originate from the phage-originated translational initiator in the following process: (1) kasugamycin inhibits bacterial growth by interfering with translation; (2) phages infect stationary-phase bacteria and transcribe their genome, including tRNAi; (3) tRNAi of phage origin hijacks the translational machinery by competition and starts to translate phage proteins, allowing progeny release and propagation; and (4) phage replication continues while the adjacent bacterial cells are still in the stationary phase due to kasugamycin. Although the mechanism might not be exactly the same, a PAS effect has been hypothesized between gentamicin (another aminoglycoside antibiotic) and a *Staphylococcus* phage [55]. The tRNAs of jumbo phages increase phage fitness by improving the translational efficiency or independence of translation from the host factors [56]. Thus, the combination of phages encoding tRNAi and kasugamycin should be included as a biocontrol agent against fire blight.

Considering the findings of a previous report elucidating the importance of administration order in devising combined treatments with phages and antibiotics [57], the next step would be optimization of the administration order with the concentrations obtained in the present study (8 log plaque forming unit [PFU]/mL and sub-MIC of kasugamycin). We proposed the use of PAS for optimizing strategies to control *E. amylovora* and, consequently, fire blight and strategies involving PAS can reduce the excessive use of antibiotics in fire blight control. This can minimize the emergence and spread of antibiotic resistance among opportunistic pathogens present in the environment [58]. We propose our phage cocktail, and its combination with kasugamycin, to be an effective protocol to control the current blight outbreaks caused by *Erwinia* in South Korea, as the pathogens tested in our study are recently recovered strains from diseased plant tissue obtained from locations across South Korea. Further studies investigating the synergistic mechanisms of kasugamycin, and phages having their own translational initiator, are expected to broaden our options for alternative antibacterial strategies and reduce the excessive use of antibiotic agents.

## 4. Materials and Methods

### 4.1. Phage Isolation

A total of 220 samples were collected comprising 94 soil samples and 126 water samples from the area affected by the fire blight outbreak in South Korea, and the phages infecting *E. amylovora* were isolated from the samples using a protocol described in previous studies [59,60]. The *Erwinia amylovora* TS3128 strain, a reference strain for research in Korea, was cultured with exponential growth, and the samples were added to the cultures in a one-to-one ratio. The mixed samples were cultured at 27 °C for 24 h to amplify the phages. Samples presenting plaques were identified, collected, and subsequently filtered through a 0.45 μm syringe filter. The double-layer agar (DLA) method was used to confirm the bacteriolysis induced by the phages [61]. Cloning of the phages from the plaques was carried out five times to purify and isolate the respective phages.

### 4.2. Phage Propagation and Purification

The DLA method was used to amplify the phages, based on a protocol described in a previous publication [62]. The top agar layer was collected in an SM buffer (50 mM Tris [pH 7.5], 100 mM NaCl, and 10 mM MgSO_4_) and mixed for 1 h. The mixture was centrifuged, and the supernatant was filtered through a 0.45 μm syringe filter to eliminate contaminants. Then, a polyethylene glycol/NaCl solution was added to the sample to precipitate the phage particles. The cesium chloride (CsCl) density gradient centrifugation method was used to purify the phage particles [45]. Phage samples with gradient layers of CsCl solution were ultracentrifuged for 3 h at 50,000× *g* using a Type 70 Ti fixed-angle titanium rotor (Beckman, Brea, CA, USA). The sedimentation bands were collected and dialyzed using a 7000 MWCO Slide-A-Lyzer^®^ Dialysis Cassette (Thermo Scientific, Waltham, MA, USA). The purified samples (>10^10^ PFU/mL) were stored at 4 °C for further analysis.

### 4.3. Transmission Electron Microscopy

The purified phage samples were attached for 1 min on separate glow-discharged TEM Grid FCF200-CU-50 Formvar/Carbon grids (Sigma-Aldrich, Burlington, MA, USA). After removing the sample solution, 2% phosphotungstic acid was added to the grids to stain the phages for 30 s, and the remaining solution was eliminated. The grids were air-dried for 1 h, and morphological study of the phages was performed using a Talos L120C transmission electron microscope (FEI, Hillsboro, OR, USA) operated at 120 kV. Three isolated virions were measured, and the mean size of the phages was calculated.

### 4.4. Bacteriophage Screening Assay

Bacteriophages were screened in two stages to select five effective phages based on their growth inhibition potential. Growth inhibition was determined based on optical density (OD) at 600 nm after 24 h of phage–bacteria co-culture. The initial screening was performed at 10^5^ CFU/mL, and the second screening was performed with 10^6^ CFU/mL of *E. amylovora*. The tests were performed in a 96-well plate with 10^8^ PFU/mL of each phage and incubated at 27 °C with shaking (150 rpm). The phages and bacteria were prepared in nutrient broth. The growth inhibition was calculated as follows:

(1) $$\%\;\mathrm{growth}=\frac{\mathrm{OD}600\;\mathrm{of}\;\mathrm{challenge}}{\mathrm{OD}600\;\mathrm{of}\;\mathrm{untreated}\;\mathrm{host}}\times 100$$

### 4.5. Bacteriophage Host Range Assay

A total of 94 strains of *E. amylovora* and 25 strains of *E. pyrifoliae* were tested to identify the host infectivity of the selected five phages: φ27, φ31, φ32, φ47, and φ48. The infectivity of phages was determined by performing a spot assay against recently recovered strains obtained from diseased plant tissue in South Korea. Serial dilutions of phage lysate (10 μL) at a concentration of 10^1^ to 10^8^ PFU/mL were added dropwise on the bacterial lawns, and the infectivity was represented as the efficiency of plating (EOP) value. The protocol was described in a previous study, with minor modifications, i.e., using a 52 °C water bath instead of a 46 °C heating block [63].

### 4.6. Bacterial Killing Assay In Vitro

The bactericidal efficacy of individual phages, and of their cocktail, was examined using *E. amylovora* TS3128 according to a method described in a previous publication, with minor modifications [64]. The strain (10^5^ CFU/mL) was infected with phages at a concentration of 10^8^ PFU/mL. The cocktail comprised identical ratios (1:1:1:1:1) of 2 × 10^7^ PFU/mL of each phage. The mixtures were cultured at 27 °C with shaking (150 rpm), and the cell counts were observed over time. Each experiment was performed in triplicates (*n* = 3).

### 4.7. Phage Resistance Assay

The phage resistance assay was performed as previously described, with minor modifications [31]. After the in vitro bacterial killing assay, the surviving colonies were sub-cultured thrice to remove the residual phages. Then, phage susceptibility was tested as described above. If plaques were not observed, the strain was confirmed to be phage resistant. Phage-resistant strains were designated as follows; φ27-resistant strain (R27), φ31-resistant strain (R31), φ32-resistant strain (R32), φ47-resistant strain (R47), and φ48-resistant strain (R48). The susceptibility of phages was determined using the five phages and the cocktail at a concentration of 2 × 10^9^ PFU/mL. Ten microliters of serial dilutions (10^−1^ to 10^−8^) of phage solution were spotted on each phage-resistant strain: R27, R31, R32, R47, and R48. Negative control (N) and wild type (WT) were also tested.

### 4.8. Minimum Inhibitory Concentration (MIC) Assay

The MIC value of kasugamycin against the wild type *E. amylovora* and phage-resistant strains was determined using the broth microdilution method [65]. Serial dilutions (two-fold) starting with 512 μg/mL were inoculated with the same volume of the bacterial solution (2 × 10^5^ CFU/mL) and incubated for 24 h at 27 °C. The MIC of the antibiotics was determined by measuring the OD at 600 nm in triplicates (*n* = 3). The growth inhibition was calculated as follows and the results were visualized in a heatmap:

(2) $$\%\;\mathrm{growth}=\frac{\mathrm{OD}600\;\mathrm{of}\;\mathrm{challenge}}{\mathrm{OD}600\;\mathrm{of}\;\mathrm{untreated}\;\mathrm{host}}\times 100$$

### 4.9. Phage–Antibiotic Synergy Assay

The advanced effect between the phage cocktail and kasugamycin was determined using *E. amylovora* with a method described in a previous study [64]. The phage cocktail comprised a five-phage mixture having each phage in the same ratio and was mixed with kasugamycin solutions diluted in nutrient broth at MIC, 1/2 MIC,1/4 MIC, and 0 MIC. The wild-type strain (10^5^ CFU/mL) was co-cultured with a phage cocktail (10^8^ PFU/mL) with or without a combination of antibiotics. The mixtures were cultured at 27 °C with shaking (150 rpm), and the cell counts were observed over time. Each experiment was performed in triplicates (*n* = 3).

### 4.10. Experiment on Apple Fruit under Controlled Conditions

Immature apples (cv. Fuji) were surface sterilized using ethanol, wounded, and infected with 2 × 10^5^ CFU/mL of *E. amylovora* TS3128 according to a method described in a previous publication [66]. Wounded fruits were administered 2 × 10^8^ PFU/mL of phages, antibiotics, or a phage–antibiotic combination and incubated in a humidified chamber at 27 °C. Symptoms were recorded at 2, 4, and 6 days after administration. The infected fruits were homogenized in order to enumerate the bacterial counts and the assay was repeated three times with three biological replicates (*n* = 3).

### 4.11. Stability Assay

The stability of the phages at different temperatures and pH conditions was examined. The phages (~1 × 10^8^ PFU/mL) were incubated at 4 (control), 20, 30, 40, and 50 °C for thermal stability. The phages (~1 × 10^8^ PFU/mL) were incubated in an SM buffer with a pH adjusted to 4, 5, 6, 7 (control), 8, and 9 using NaOH or HCl at 27 °C for the pH stability assay. After incubation for 60 min, the sample concentrations were evaluated in triplicates (*n* = 3). The stability value was standardized by using control as 100%.

### 4.12. DNA Isolation and Sequencing

The conventional phenol–chloroform method was used to isolate DNA from the phages [67]. RNase A (10 IU), DNase I (10 IU), and 10X DNase I buffer (Takara Bio, Kusatsu, Japan) were added to 1 mL of the phage solution of 10^10^ PFU/mL, and then the solution was incubated at 37 °C for 1 h. Fifty microliters of 0.5 M ethylenediaminetetraacetic acid and proteinase K were added in the solution to inactivate the enzymes and hydrolyze the proteins, respectively. A mixture of isoamyl alcohol, chloroform, and phenol (1:24:25) was added, and the solution was centrifuged. Ethanol was added to the solution and the supernatant was removed. The precipitate was then resuspended in distilled water. The phage DNA was sequenced using an ABI 3730xl System (Thermo Fisher Scientific, Waltham, MA, USA) at Macrogen (Seoul, South Korea). FastQC (v0.11.6) was used to check the read quality. Trimmomatic (v0.36) was used to remove adapter sequences, and the assembly was performed using SPAdes (v3.12).

### 4.13. Genome Analysis

GenMarkS, Prokka (v1.12b), Nucleotide BLAST, and HHpred were used for gene prediction and annotation [68,69,70,71]. Identification of tRNA was conducted using tRNAscan-SE (v2.0) [72]. Visualization of the genome was conducted using DNAPlotter [73]. The genome dot plot was created using Gepard with default settings [74]. Phylogenetic analysis was performed using VICTOR [75]. In VICTOR, 79 phages infecting *Erwinia* spp., *Dickeya* spp., *Pantoea* spp., and *Pectobacterium* spp. were analyzed using default settings. Alignment with progressive Mauve was used for φ27, φ31, φ32, φ47, φ48, *Pantoea* phage vB_PagM_SSEM1, *Dickeya* phage vB_DsoM_AD1, *Erwinia* phage vB_EamM_Alexandra, *Pectobacterium* phage CBB, and *Erwinia* phage vB_EamM_RAY for the comparative genomic analysis [76]. The comparable phages were selected based on their genomic closeness with the five phages used in this study. The result was visualized with the default settings.

### 4.14. Statistical Analysis

Each experimental set of data of in vitro bacterial killing assay, phage–antibiotic synergy assay, and experiments on apple fruit under controlled conditions was statistically analyzed with one-way analysis of variance (ANOVA) and the Tukey post-hoc test using SigmaPlot software version 12.5 (Systat Software, San Jose, CA, USA).

## Figures and Tables

**Table 1:** General features of genomes of *Erwinia* phages pEa_SNUABM_27, pEa_SNUABM_31, pEa_SNUABM_32, pEa_SNUABM_47, and pEa_SNUABM_48.

Bacterio-Phage	pEa_SNUABM_27	pEa_SNUABM_31	pEa_SNUABM_32	pEa_SNUABM_47	pEa_SNUABM_48
Genus	*Loessnervirus*	*Alexandravirus*	*Alexandravirus*	*Eneladusvirus*	unclassified
Size (bp)	53,014	265,765	265,891	355,376	294,405
Open reading frames (ORFs)	78	337	336	540	358
tRNAs	1	0	0	35	2
Guanine–cytosine (GC) content (%)	44.07	49.53	49.19	34.48	49.52
DNA Circularity	Circular	Circular	Circular	Circular	Circular
Accession number	MW349138.1	MZ443773.1	MZ443774.1	MT939487.1	MW879340.1
Capsid diameter (nm)	68.5 ± 2.76	139.15 ± 5.47	130.03 ± 6.06	127.74 ± 6.58	139.74 ± 2.34
Tail length (nm)	115.1 ± 2.16	196.32 ± 11.45	168.88 ± 6.53	126.61 ± 2.93	150.35 ± 16.91

## Data Availability

All data generated or analyzed during this study are included in this published article.

## Supplementary Materials

The following supporting information can be downloaded at: https://www.mdpi.com/article/10.3390/antibiotics11111566/s1, Figure S1: Host range of *Erwinia* phages φ27, φ31, φ32, φ47, and φ48, Figure S2: Stability of the phage virions, TableS1: Screening assay of the bacteriophages, Table S2: Functional classification of ORFs in *Erwinia* phage pEa_SNUABM_27, Table S3: Functional classification of ORFs in *Erwinia* phage pEa_SNUABM_31, Table S4: Functional classification of ORFs in *Erwinia* phage pEa_SNUABM_32, Table S5: Functional classification of ORFs in *Erwinia* phage pEa_SNUABM_47, Table S6: Functional classification of ORFs in *Erwinia* phage pEa_SNUABM_48.

## Abbreviations

MIC	minimal inhibitory concentration
phages	bacteriophages
CFU	colony forming unit
ORFs	open reading frames
WT	wild type
PAS	phage–antibiotic synergy
GC	guanine–cytosine
PFU	plaque forming unit
DLA	double layer agar
CsCl	cesium chloride
OD	optical density
EOP	efficiency of plating
VICTOR	Virus Classification and Tree Building Online Resource
ANOVA	analysis of variance
