# Supplementary material for: Phage Cocktail in Combination with Kasugamycin as a Potential Treatment for Fire Blight Caused by Erwinia amylovora
Source: Antibiotics (Basel). 2022 Nov 6;11(11):1566. doi: 10.3390/antibiotics11111566 (PMC9686651; doi:10.3390/antibiotics11111566)
Supplement: Supplementary file 1 [file antibiotics-11-01566-s001.zip › antibiotics-1958277-supplementary.pdf]

Figure S1. Host range of *Erwinia* phages φ27, φ31, φ32, φ47, and φ48. In total, 94 strains of *E. amylovora* and 25 strains of *E. pyrifoliae* were tested as hosts for the phages.

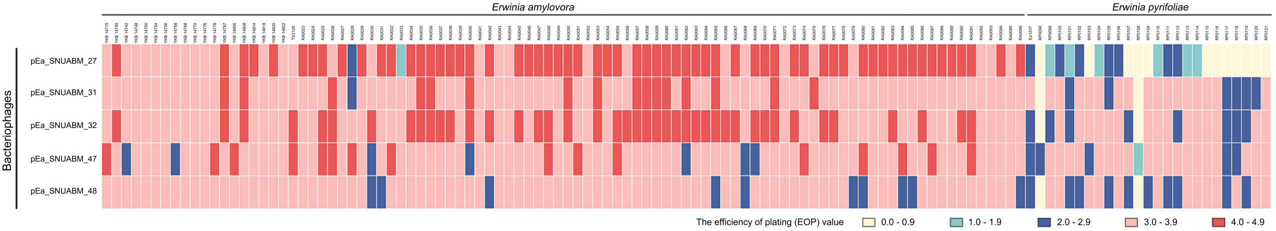

Figure S2. Stability of the phage virions (a) φ27, (c) φ31, (e) φ32, (g) φ47, and (i) φ48 for a range of thermal, and (b) φ27, (d) φ 31, (f) φ32, (h) φ47, and (j) φ48 for a range of pH conditions. PFU means plaque-forming unit.

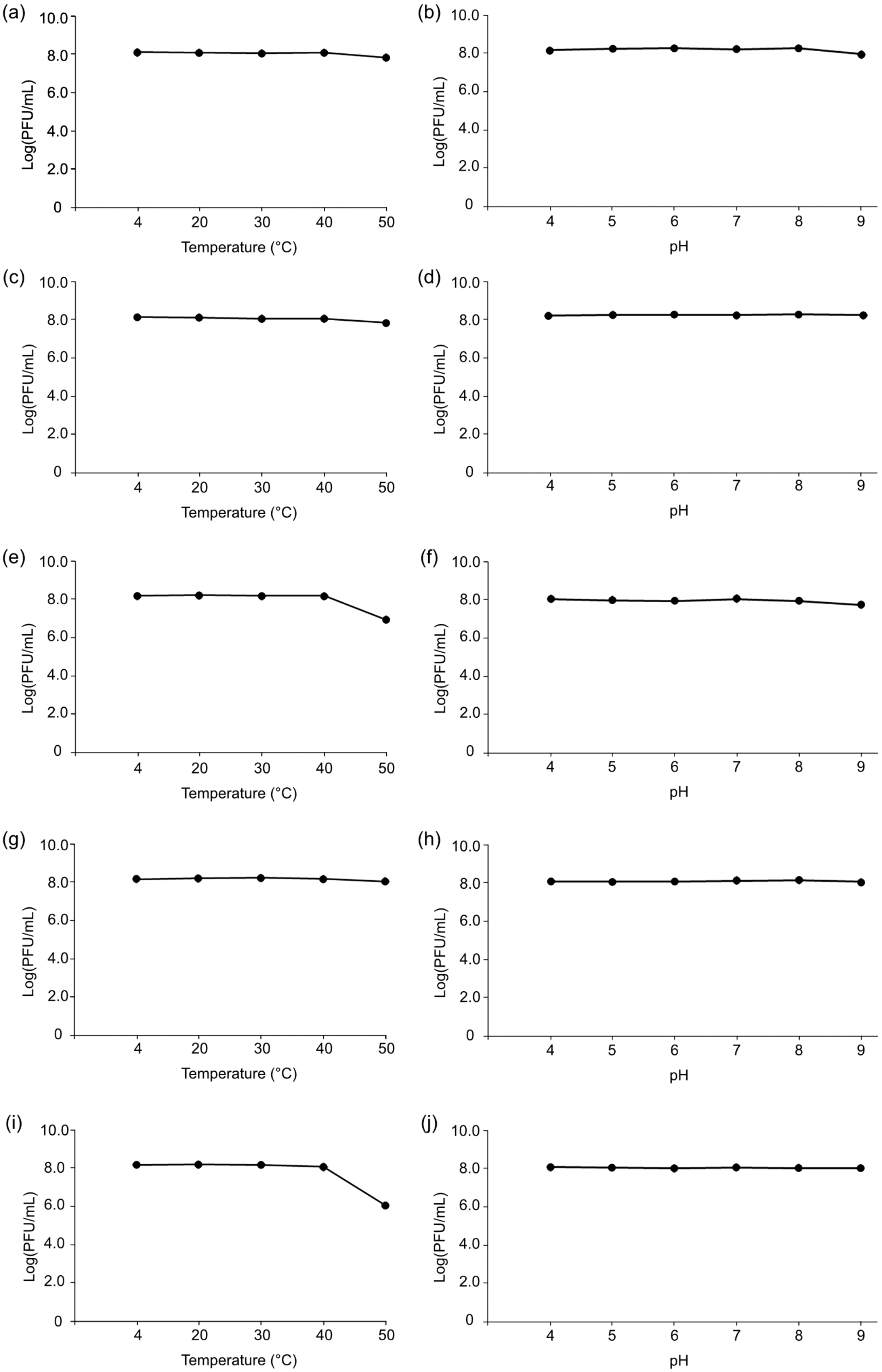

Table S1. Screening assay of the bacteriophages. The table shows the allocated bacteriophages for each slot at each screening assay (a). Graphs show the standardized growth (%) observed on 24 h co-culture (b, c). The first screening was performed with bacteria at inoculum concentration of 5 LogCFU/mL and the selected phages were marked with yellow shade (b). Second screening was performed with bacteria at inoculum concentration of 6 LogCFU/mL and the selected phages were marked with green shade (c).

(a)

φ1	φ9	φ17	φ25	φ33	φ41	φ49
φ2	φ10	φ18	φ26	φ34	φ42	φ50
φ3	φ11	φ19	φ27	φ35	φ43	φ51
φ4	φ12	φ20	φ28	φ36	φ44	φ52
φ5	φ13	φ21	φ29	φ37	φ45	φ53
φ6	φ14	φ22	φ30	φ38	φ46	φ54
φ7	φ15	φ23	φ31	φ39	φ47	Control
φ8	φ16	φ24	φ32	φ40	φ48	

(b)

11.32±0.24	13.69±1.08	30.02±11.7	11.08±0.95	11.14±0.22	12.40±0.03	10.16±0.38
10.90±1.07	11.04±0.52	9.82±0.48	10.46±0.44	11.41±0.76	10.04±0.82	10.35±0.87
14.13±1.85	13.17±1.80	11.15±1.49	10.74±0.50	11.03±0.29	10.96±0.98	11.43±2.00
11.81±0.53	20.57±5.32	10.55±0.32	10.53±0.55	10.58±0.29	10.60±1.32	10.37±0.11
21.15±5.78	12.98±0.14	12.90±0.69	12.31±0.39	11.69±0.34	11.18±0.97	11.86±1.39
10.67±0.50	10.64±0.11	11.11±0.91	10.28±0.99	10.13±0.51	10.65±0.76	10.62±0.46
12.33±4.56	10.09±0.51	10.99±1.53	10.13±0.38	39.95±0.72	10.80±0.38	100
11.14±0.45	10.19±0.75	11.18±0.92	9.82±0.59	10.37±0.39	10.65±0.52	

(c)

9.6±0.98	8.57±1.11	14.50±3.59	9.06±0.80	9.12±0.42	9.55±1.65	8.91±0.56
9.21±0.97	9.79±1.17	8.63±0.31	11.60±0.62	8.28±0.22	8.20±0.53	9.16±1.55
9.21±0.31	9.43±1.02	10.37±1.29	3.95±2.44	9.08±0.13	8.52±0.10	8.33±0.63
10.64±1.02	14.26±3.55	10.51±0.96	8.76±0.58	10.32±2.56	8.61±0.76	8.42±0.38
13.55±1.75	12.00±1.45	11.41±1.37	9.37±0.48	9.29±0.29	9.90±0.81	9.31±0.63
9.03±0.61	12.63±3.28	9.41±1.53	8.04±0.46	9.80±0.54	8.78±0.55	8.43±2.52
11.58±2.89	10.09±1.68	8.65±0.27	7.86±0.10	36.84±2.95	7.96±0.21	100
8.69±0.31	9.57±0.91	10.95±2.26	7.89±0.29	12.52±3.66	7.80±0.22	

Table S2. Functional classification of ORFs in *Erwinia* phage pEa_SNUABM_27.

Group	Locus tag	Encoded protein	Related organism	Query cover (%)	Identity (%)
Nucleotide metabolism	pEa_SNUABM27_00001	putative RNA polymerase	*Pantoea* phage vB_PagM_SSEM1	99	94.14
Nucleotide metabolism	pEa_SNUABM27_00013	putative single-stranded DNA binding protein	*Pantoea* phage vB_PagM_SSEM1	99	73.23
Lysis	pEa_SNUABM27_00021	putative L-alanyl-D-glutamate peptidase	*Pantoea* phage vB_PagM_SSEM1	99	97.73
Nucleotide metabolism	pEa_SNUABM27_00024	putative ArdA antirestriction protein	*Erwinia* phage vB_EamM-Y2	98	48.11
tRNA	pEa_SNUABM27_00033	tRNA			
Additional function	pEa_SNUABM27_00038	putative thymidylate synthase	*Erwinia* phage vB_EamM-Y2	99	79.72
Nucleotide metabolism	pEa_SNUABM27_00039	putative DNA helicase	*Pectobacterium* phage PP101	99	82.25
Nucleotide metabolism	pEa_SNUABM27_00040	putative DNA polymerase	*Erwinia* phage vB_EamM-Y2	99	87.42
Nucleotide metabolism	pEa_SNUABM27_00043	putative exonuclease	*Erwinia* phage vB_EamM-Y2	99	83.73
Nucleotide metabolism	pEa_SNUABM27_00045	putative DNA ligase	*Erwinia* phage vB_EamM-Y2	99	87.4
Nucleotide metabolism	pEa_SNUABM27_00046	putative deoxynucleoside monophosphate kinase	*Pantoea* phage vB_PagM_SSEM1	99	89.71
Nucleotide metabolism	pEa_SNUABM27_00047	putative deoxyuridine 5'-triphosphate nucleotidohydrolase	*Erwinia* phage vB_EamM-Y2	99	89.93
Nucleotide metabolism	pEa_SNUABM27_00052	putative terminase large subunit	*Pantoea* phage vB_PagM_SSEM1	99	91.93
Structure & packaging	pEa_SNUABM27_00054	putative portal protein	*Erwinia* phage vB_EamM-Y2	96	87.59
Structure & packaging	pEa_SNUABM27_00057	putative major capsid protein	*Pantoea* phage vB_PagM_SSEM1	99	90.77
Structure & packaging	pEa_SNUABM27_00060	putative head-tail adaptor	*Pantoea* phage vB_PagM_SSEM1	99	77.5
Structure & packaging	pEa_SNUABM27_00061	putative tail completion protein	*Pantoea* phage vB_PagM_SSEM1	99	84.12
Structure & packaging	pEa_SNUABM27_00062	putative tail sheath protein	*Erwinia* phage vB_EamM-Y2	99	76.6
Structure & packaging	pEa_SNUABM27_00063	putative tail tube protein	*Erwinia* phage vB_EamM-Y2	99	94.04
Structure & packaging	pEa_SNUABM27_00064	putative tail assembly chaperone protein	*Pantoea* phage vB_PagM_SSEM1	99	83.97
Additional function	pEa_SNUABM27_00065	phage enzyme	*Erwinia* phage vB_EamM-Y2	99	65.1
Structure & packaging	pEa_SNUABM27_00066	Putative baseplate hub protein	*Erwinia* phage vB_EamM-Y2	96	64.54
Structure & packaging	pEa_SNUABM27_00069	putative baseplate assembly protein	*Erwinia* phage vB_EamM-Y2	99	73.54
Structure & packaging	pEa_SNUABM27_00070	putative baseplate wedge protein	*Erwinia* phage vB_EamM-Y2	98	72.88
Structure & packaging	pEa_SNUABM27_00071	putative baseplate wedge protein	*Pantoea* phage vB_PagM_SSEM1	99	75.13
Structure & packaging	pEa_SNUABM27_00072	putative basepate protein	*Pantoea* phage vB_PagM_SSEM1	99	80.93
Lysis	pEa_SNUABM27_00076	putative holin	*Pantoea* phage vB_PagM_SSEM1	93	91.23
Lysis	pEa_SNUABM27_00077	putative endolysin	*Erwinia* phage vB_EamM-Y2	98	63.98

Table S3. Functional classification of ORFs in *Erwinia* phage pEa_SNUABM_31.

Group	Locus tag	Encoded protein	Related organism	Query cover (%)	Identity (%)
Nucleotide metabolism	pEa_SNUABM31_00001	putative DNA helicase	*Dickeya* phage vB_DsoM_AD1	99	96.63
Structure & packaging	pEa_SNUABM31_00004	putative terminase large subunit	*Dickeya* phage vB_DsoM_AD1	99	90.68
Nucleotide metabolism	pEa_SNUABM31_00005	putative RNA polymerase	*Erwinia* phage vB_EamM_Alexandra	98	81.69
Structure & packaging	pEa_SNUABM31_00006	putative portal protein	*Erwinia* phage vB_EamM_Alexandra	99	95.34
Nucleotide metabolism	pEa_SNUABM31_00011	putative endonuclease	*Dickeya* phage vB_DsoM_AD1	99	82.46
Nucleotide metabolism	pEa_SNUABM31_00013	putative O-acetyl-ADP-ribose deacetylase	*Dickeya* phage vB_DsoM_AD1	98	87.31
Nucleotide metabolism	pEa_SNUABM31_00016	putative methyltransferase	*Dickeya* phage vB_DsoM_AD1	99	91.41
Nucleotide metabolism	pEa_SNUABM31_00017	putative glycosyl transferase	*Myoviridae* sp.	53	29
Additional function	pEa_SNUABM31_00020	putative asparagine synthase	*Dickeya* phage vB_DsoM_AD1	99	85.47
Nucleotide metabolism	pEa_SNUABM31_00025	putative DNA primase	*Erwinia* phage vB_EamM_Deimos_ Minion	98	83.95
Nucleotide metabolism	pEa_SNUABM31_00026	putative ATP-binding cassette transporter	*Dickeya* phage vB_DsoM_AD1	98	88.11
Nucleotide metabolism	pEa_SNUABM31_00029	putative DNA adenine methylase	*Dickeya* phage vB_DsoM_AD1	99	95.71
Nucleotide metabolism	pEa_SNUABM31_00036	putative cytidine monophosphate deaminase	*Dickeya* phage vB_DsoM_AD1	99	86.13
Additional function	pEa_SNUABM31_00046	putative thymidylate synthase	*Dickeya* phage vB_DsoM_AD1	99	95.34
Nucleotide metabolism	pEa_SNUABM31_00050	putative transcriptional repressor	*Erwinia* phage vB_EamM_Alexandra	99	90.25
Nucleotide metabolism	pEa_SNUABM31_00051	putative DNA-cytosine methyltransferase	*Dickeya* phage vB_DsoM_AD1	99	85.19
Nucleotide metabolism	pEa_SNUABM31_00067	putative glutamyl transpeptidase pyrophosphokinase	*Dickeya* phage vB_DsoM_AD1	99	92.41
Lysis	pEa_SNUABM31_00075	putative spanin	*Erwinia* phage vB_EamM_Y3	99	83.12
Additional function	pEa_SNUABM31_00102	putative ATP-binding cassette transporter	Dickeya phage vB_DsoM_AD1	99	86.81
Structure & packaging	pEa_SNUABM31_00105	putative tail fiber protein	*Dickeya* phage vB_DsoM_AD1	99	96.34
Structure & packaging	pEa_SNUABM31_00107	putative long tail fiber protein	*Dickeya* phage vB_DsoM_AD1	99	88.74
Structure & packaging	pEa_SNUABM31_00109	putative tail fiber protein	*Erwinia* phage vB_EamM_Y3	99	78.53
Structure & packaging	pEa_SNUABM31_00110	putative tail fiber protein	*Dickeya* phage vB_DsoM_AD1	99	91.43
Structure & packaging	pEa_SNUABM31_00111	putative tail protein	*Dickeya* phage vB_DsoM_AD1	99	84.58
Structure & packaging	pEa_SNUABM31_00114	putative baseplate wedge subunit protein	*Dickeya* phage vB_DsoM_AD1	99	95.07
Structure & packaging	pEa_SNUABM31_00115	putative baseplate protein	*Dickeya* phage vB_DsoM_AD1	99	94.29
Structure & packaging	pEa_SNUABM31_00116	putative baseplate spike protein	*Dickeya* phage vB_DsoM_AD1	98	95.83
Nucleotide metabolism	pEa_SNUABM31_00120	putative dTMP kinase	*Dickeya* phage vB_DsoM_AD1	98	89.9
Nucleotide metabolism	pEa_SNUABM31_00121	putative MmcB-like DNA repair protein	*Erwinia* phage vB_EamM_Alexandra	99	94.74
Nucleotide metabolism	pEa_SNUABM31_00122	putative NUDIX hydrolyase	*Dickeya* phage vB_DsoM_AD1	99	85.63
Structure & packaging	pEa_SNUABM31_00126	putative baseplate protein	*Dickeya* phage vB_DsoM_AD1	99	91.6

Structure & packaging	pEa_SNUABM31_00127	putative baseplate hub protein	*Erwinia* phage vB_EamM_Alexandra	99	87.96
Nucleotide metabolism	pEa_SNUABM31_00128	putative transcriptional coactivator	*Dickeya* phage vB_DsoM_AD1	99	83.33
Structure & packaging	pEa_SNUABM31_00129	putative baseplate wedge protein	*Dickeya* phage vB_DsoM_AD1	98	80.41
Structure & packaging	pEa_SNUABM31_00130	putative contractile injection system tube protein	*Dickeya* phage vB_DsoM_AD1	98	92.16
Structure & packaging	pEa_SNUABM31_00131	putative tape measure protein	*Dickeya* phage vB_DsoM_AD1	99	75.05
Structure & packaging	pEa_SNUABM31_00132	putative baseplate hub assembly protein	*Erwinia* phage vB_EamM_Alexandra	99	85.36
Structure & packaging	pEa_SNUABM31_00133	putative baseplate hub assembly protein	*Dickeya* phage vB_DsoM_AD1	99	90.31
Structure & packaging	pEa_SNUABM31_00134	putative tail tube protein	*Dickeya* phage vB_DsoM_AD1	99	95.88
Structure & packaging	pEa_SNUABM31_00135	putative tail tube protein	*Dickeya* phage vB_DsoM_AD1	99	91.96
Structure & packaging	pEa_SNUABM31_00136	putative tail sheath protein	*Dickeya* phage vB_DsoM_AD1	99	98.82
Structure & packaging	pEa_SNUABM31_00137	putative tail sheath protein	*Dickeya* phage vB_DsoM_AD1	99	93.05
Structure & packaging	pEa_SNUABM31_00141	putative major capsid protein	*Dickeya* phage vB_DsoM_AD1	99	91.53
Structure & packaging	pEa_SNUABM31_00142	putative structural protein	*Dickeya* phage vB_DsoM_AD1	99	90.64
Nucleotide metabolism	pEa_SNUABM31_00144	putative M34 peptidase	*Dickeya* phage vB_DsoM_AD1	99	76.82
Structure & packaging	pEa_SNUABM31_00145	putative prohead core protease	*Dickeya* phage vB_DsoM_AD1	99	96.14
Nucleotide metabolism	pEa_SNUABM31_00148	putative glycosyl transferase	*Dickeya* phage vB_DsoM_AD1	95	81.5
Nucleotide metabolism	pEa_SNUABM31_00150	putative DNA ligase	*Dickeya* phage vB_DsoM_AD1	99	92.44
Structure & packaging	pEa_SNUABM31_00156	putative major tail protein	*Dickeya* phage vB_DsoM_AD1	99	90.72
Structure & packaging	pEa_SNUABM31_00160	putative tail sheath protein	*Dickeya* phage vB_DsoM_AD1	99	92.83
Nucleotide metabolism	pEa_SNUABM31_00161	putative exonuclease	*Dickeya* phage vB_DsoM_AD1	99	81.99
Nucleotide metabolism	pEa_SNUABM31_00170	putative DNA repair helicase	*Dickeya* phage vB_DsoM_AD1	99	97.56
Nucleotide metabolism	pEa_SNUABM31_00173	putative DNA polymerase III	*Dickeya* phage vB_DsoM_AD1	99	91.84
Nucleotide metabolism	pEa_SNUABM31_00175	putative DNA polymerase I	*Dickeya* phage vB_DsoM_AD1	99	94.21
Nucleotide metabolism	pEa_SNUABM31_00178	putative ATP-dependent DNA helicase	*Dickeya* phage vB_DsoM_AD1	99	91.22
Nucleotide metabolism	pEa_SNUABM31_00181	putative HNH endonuclease	*Dickeya* phage vB_DsoM_AD1	99	92.56
Structure & packaging	pEa_SNUABM31_00184	putative head-to-tail joining protein	*Dickeya* phage vB_DsoM_AD1	99	93.54
Nucleotide metabolism	pEa_SNUABM31_00188	putative recombination-related endonuclease	*Dickeya* phage vB_DsoM_AD1	99	91.57
Nucleotide metabolism	pEa_SNUABM31_00190	putative ssDNA binding protein	*Dickeya* phage vB_DsoM_AD1	98	89.29
Lysis	pEa_SNUABM31_00197	putative glycosyl hydrolase	*Erwinia* phage vB_EamM_Alexandra	99	94.74
Nucleotide metabolism	pEa_SNUABM31_00203	putative exonuclease	*Erwinia* phage vB_EamM_Alexandra	99	84.07
Nucleotide metabolism	pEa_SNUABM31_00209	putative DNA polymerase III	*Dickeya* phage vB_DsoM_AD1	94	92.25
Nucleotide metabolism	pEa_SNUABM31_00211	putative deoxyribonucleotidase	*Erwinia* phage vB_EamM_Alexandra	99	84.62
Nucleotide metabolism	pEa_SNUABM31_00214	putative M34 peptidase	*Dickeya* phage vB_DsoM_AD1	99	90.43
Nucleotide metabolism	pEa_SNUABM31_00219	putative helicase	*Erwinia* phage vB_EamM_Alexandra	97	52.61
Structure & packaging	pEa_SNUABM31_00222	putative holliday junction resolvase	*Erwinia* phage vB_EamM_Alexandra	99	90.66
Nucleotide metabolism	pEa_SNUABM31_00228	putative DNA primase	*Dickeya* phage vB_DsoM_AD1	99	97.43

Nucleotide metabolism	pEa_SNUABM31_00230	putative exonuclease	*Dickeya* phage vB_DsoM_AD1	99	94.46
Nucleotide metabolism	pEa_SNUABM31_00239	putative cyclic phosphodiesterase	*Dickeya* phage vB_DsoM_AD1	99	90.96
Nucleotide metabolism	pEa_SNUABM31_00242	putative RpoD subfamily RNA polymerase sigma factor	*Dickeya* phage vB_DsoM_AD1	99	95.35
Nucleotide metabolism	pEa_SNUABM31_00243	putative ssDNA binding protein	*Dickeya* phage vB_DsoM_AD1	63	97.76
Nucleotide metabolism	pEa_SNUABM31_00244	putative replicative DNA helicase	*Dickeya* phage vB_DsoM_AD1	99	93.48
Nucleotide metabolism	pEa_SNUABM31_00267	putative acetyltransferase	*Dickeya* phage vB_DsoM_AD1	99	88.89
Nucleotide metabolism	pEa_SNUABM31_00271	putative RNA 2'-phosphotransferase	*Dickeya* phage vB_DsoM_AD1	99	82.49
Nucleotide metabolism	pEa_SNUABM31_00273	putative radical S-adenosyl-L-methionine protein	*Erwinia* phage vB_EamM_Alexandra	96	80.64
Nucleotide metabolism	pEa_SNUABM31_00277	putative DksA/TraR family C4-type zinc finger protein	*Dickeya* phage vB_DsoM_AD1	98	97.7
Nucleotide metabolism	pEa_SNUABM31_00288	putative UV damage repair endonuclease	*Dickeya* phage vB_DsoM_AD1	99	80.98
Nucleotide metabolism	pEa_SNUABM31_00299	putative dUTPase	*Dickeya* phage vB_DsoM_AD1	99	95.24
Lysis	pEa_SNUABM31_00302	putative lytic transglycosylase	*Erwinia* phage vB_EamM_Alexandra	99	87.17
Nucleotide metabolism	pEa_SNUABM31_00304	putative metal-dependent phosphohydrolase	*Erwinia* phage vB_EamM_Y3	99	58.92
Nucleotide metabolism	pEa_SNUABM31_00330	putative DNA gyrase subunit B	*Dickeya* phage vB_DsoM_AD1	97	94.86
Nucleotide metabolism	pEa_SNUABM31_00332	Putative DNA gyrase subunit A	*Erwinia* phage vB_EamM_Y3	97	84.01
Nucleotide metabolism	pEa_SNUABM31_00334	putative endonuclease	*Erwinia* phage vB_EamM_Alexandra	99	88.83

Table S4. Functional classification of ORFs in *Erwinia* phage pEa_SNUABM_32.

Group	Locus tag	Encoded protein	Related organism	Query cover (%)	Identity (%)
Nucleotide metabolism	pEa_SNUABM32_00001	putative DNA helicase	*Dickeya* phage vB_DsoM_AD1	99	96.22
Nucleotide metabolism	pEa_SNUABM32_00004	putative terminase large subunit	*Dickeya* phage vB_DsoM_AD1	99	94.37
Nucleotide metabolism	pEa_SNUABM32_00005	putative RNA polymerase	*Erwinia* phage vB_EamM_Alexandra	98	85.92
Structure & packaging	pEa_SNUABM32_00006	putative portal protein	*Dickeya* phage vB_DsoM_AD1	99	96.29
Nucleotide metabolism	pEa_SNUABM32_00011	putative endonuclease	*Erwinia* phage vB_EamM_Alexandra	99	84.5
Nucleotide metabolism	pEa_SNUABM32_00013	putative O-acetyl-ADP-ribose deacetylase	*Dickeya* phage vB_DsoM_AD1	98	87.31
Nucleotide metabolism	pEa_SNUABM32_00016	putative methyltransferase	*Dickeya* phage vB_DsoM_AD1	99	91.02
Additional function	pEa_SNUABM32_00020	putative asparagine synthase	*Dickeya* phage vB_DsoM_AD1	99	85.47
Nucleotide metabolism	pEa_SNUABM32_00025	putative DNA primase	*Erwinia* phage vB_EamM_Deimos_Minion	98	83.95
Nucleotide metabolism	pEa_SNUABM32_00026	putative ATP-binding cassette transporter	*Dickeya* phage vB_DsoM_AD1	98	88.11
Nucleotide metabolism	pEa_SNUABM32_00028	putative DNA adenine methylase	*Dickeya* phage vB_DsoM_AD1	99	96.07
Nucleotide metabolism	pEa_SNUABM32_00036	putative cytidine monophosphate deaminase	*Dickeya* phage vB_DsoM_AD1	99	86.13
Additional function	pEa_SNUABM32_00046	putative thymidylate synthase	*Dickeya* phage vB_DsoM_AD1	99	95.34
Nucleotide metabolism	pEa_SNUABM32_00050	putative transcriptional repressor	*Dickeya* phage vB_DsoM_AD1	99	90.25
Nucleotide metabolism	pEa_SNUABM32_00051	putative DNA-cytosine methyltransferase	*Erwinia* phage vB_EamM_Alexandra	99	86.18
Nucleotide metabolism	pEa_SNUABM32_00067	putative glutamyl transpeptidase pyrophosphokinase	*Dickeya* phage vB_DsoM_AD1	99	93.3
Lysis	pEa_SNUABM32_00075	putative spanin	*Erwinia* phage vB_EamM_Y3	99	83.12
Nucleotide metabolism	pEa_SNUABM32_00102	putative ATP-binding cassette transporter	*Dickeya* phage vB_DsoM_AD1	99	86.45
Structure & packaging	pEa_SNUABM32_00105	putative tail fiber protein	*Dickeya* phage vB_DsoM_AD1	99	96.34
Structure & packaging	pEa_SNUABM32_00107	putative long tail fiber protein	*Dickeya* phage vB_DsoM_AD1	99	88.74
Structure & packaging	pEa_SNUABM32_00109	putative tail fiber protein	*Erwinia* phage vB_EamM_Y3	99	79.75
Structure & packaging	pEa_SNUABM32_00110	putative tail fiber protein	*Dickeya* phage vB_DsoM_AD1	99	89.52
Structure & packaging	pEa_SNUABM32_00114	putative baseplate wedge subunit protein	*Dickeya* phage vB_DsoM_AD1	99	92.81
Structure & packaging	pEa_SNUABM32_00115	putative baseplate protein	*Dickeya* phage vB_DsoM_AD1	99	95
Structure & packaging	pEa_SNUABM32_00116	putative baseplate spike protein	*Dickeya* phage vB_DsoM_AD1	98	91.67
Nucleotide metabolism	pEa_SNUABM32_00120	putative dTMP kinase	*Dickeya* phage vB_DsoM_AD1	99	93.14
Nucleotide metabolism	pEa_SNUABM32_00121	putative MmcB-like DNA repair protein	*Erwinia* phage vB_EamM_Y3	99	95.09
Nucleotide metabolism	pEa_SNUABM32_00122	putative NUDIX hydrolase	*Dickeya* phage vB_DsoM_AD1	99	95.39
Structure & packaging	pEa_SNUABM32_00126	putative baseplate protein	*Erwinia* phage vB_EamM_Y3	99	89.69
Structure & packaging	pEa_SNUABM32_00127	putative baseplate hub protein	*Erwinia* phage vB_EamM_Alexandra	99	88.59
Nucleotide metabolism	pEa_SNUABM32_00128	putative transcriptional coactivator	*Dickeya* phage vB_DsoM_AD1	99	83.33

Structure & packaging	pEa_SNUABM32_00129	putative baseplate wedge protein	*Dickeya* phage vB_DsoM_AD1	98	85.57
Structure & packaging	pEa_SNUABM32_00130	putative contractile injection system tube protein	*Dickeya* phage vB_DsoM_AD1	98	91.42
Structure & packaging	pEa_SNUABM32_00131	putative tape measure protein	*Erwinia* phage vB_EamM_Alexandra	99	77.29
Structure & packaging	pEa_SNUABM32_00132	putative baseplate hub assembly protein	*Erwinia* phage vB_EamM_Alexandra	99	88.57
Structure & packaging	pEa_SNUABM32_00133	putative baseplate hub assembly protein	*Dickeya* phage vB_DsoM_AD1	99	86.78
Structure & packaging	pEa_SNUABM32_00134	putative tail tube protein	*Erwinia* phage vB_EamM_Alexandra	99	97.06
Structure & packaging	pEa_SNUABM32_00135	putative tail tube protein	*Dickeya* phage vB_DsoM_AD1	99	95.98
Structure & packaging	pEa_SNUABM32_00136	putative tail tube protein	*Dickeya* phage vB_DsoM_AD1	99	98.82
Structure & packaging	pEa_SNUABM32_00137	putative tail sheath protein	*Dickeya* phage vB_DsoM_AD1	99	96.61
Structure & packaging	pEa_SNUABM32_00141	putative major capsid protein	*Dickeya* phage vB_DsoM_AD1	99	94.81
Structure & packaging	pEa_SNUABM32_00142	putative structural protein	*Dickeya* phage vB_DsoM_AD1	99	94.04
Nucleotide metabolism	pEa_SNUABM32_00144	putative M34 peptidase	*Dickeya* phage vB_DsoM_AD1	99	86.44
Structure & packaging	pEa_SNUABM32_00145	putative prohead core protease	*Dickeya* phage vB_DsoM_AD1	99	95.75
Nucleotide metabolism	pEa_SNUABM32_00148	putative glycosyl transferase	*Dickeya* phage vB_DsoM_AD1	95	85.5
Nucleotide metabolism	pEa_SNUABM32_00150	putative DNA ligase	*Dickeya* phage vB_DsoM_AD1	99	94.6
Structure & packaging	pEa_SNUABM32_00156	putative major tail protein	*Dickeya* phage vB_DsoM_AD1	99	90.42
Structure & packaging	pEa_SNUABM32_00160	putative tail sheath protein	*Dickeya* phage vB_DsoM_AD1	99	96.95
Nucleotide metabolism	pEa_SNUABM32_00161	putative exonuclease	*Dickeya* phage vB_DsoM_AD1	99	96.12
Nucleotide metabolism	pEa_SNUABM32_00170	putative DNA repair helicase	*Dickeya* phage vB_DsoM_AD1	99	97
Nucleotide metabolism	pEa_SNUABM32_00173	putative DNA polymerase III	*Dickeya* phage vB_DsoM_AD1	99	96.17
Nucleotide metabolism	pEa_SNUABM32_00175	putative DNA polymerase I	*Dickeya* phage vB_DsoM_AD1	99	94.21
Nucleotide metabolism	pEa_SNUABM32_00179	putative ATP-dependent DNA helicase	*Dickeya* phage vB_DsoM_AD1	99	92.26
Nucleotide metabolism	pEa_SNUABM32_00182	putative HNH endonuclease	*Dickeya* phage vB_DsoM_AD1	99	90.91
Structure & packaging	pEa_SNUABM32_00185	putative head-to-tail joining protein	*Dickeya* phage vB_DsoM_AD1	99	94.32
Nucleotide metabolism	pEa_SNUABM32_00189	putative recombination-related endonuclease	*Dickeya* phage vB_DsoM_AD1	99	93
Nucleotide metabolism	pEa_SNUABM32_00191	putative ssDNA binding protein	*Dickeya* phage vB_DsoM_AD1	95	81.33
Lysis	pEa_SNUABM32_00201	putative glycosyl hydrolase	*Dickeya* phage vB_DsoM_AD1	99	97.89
Nucleotide metabolism	pEa_SNUABM32_00207	putative exonuclease	*Dickeya* phage vB_DsoM_AD1	99	88.69
Nucleotide metabolism	pEa_SNUABM32_00211	putative ADP-ribosyltransferase	*Shigella* phage SP18	75	31.25
Nucleotide metabolism	pEa_SNUABM32_00213	putative DNA polymerase III	*Dickeya* phage vB_DsoM_AD1	93	91.77
Nucleotide metabolism	pEa_SNUABM32_00215	putative deoxyribonucleotidase	*Dickeya* phage vB_DsoM_AD1	98	92.27
Nucleotide metabolism	pEa_SNUABM32_00218	putative M34 peptidase	*Dickeya* phage vB_DsoM_AD1	99	91.98
Nucleotide metabolism	pEa_SNUABM32_00223	putative helicase	*Erwinia* phage vB_EamM_Alexandra	99	43.67
Structure & packaging	pEa_SNUABM32_00227	putative holliday junction resolvase	*Dickeya* phage vB_DsoM_AD1	99	92.86
Nucleotide metabolism	pEa_SNUABM32_00233	putative DNA primase	*Dickeya* phage vB_DsoM_AD1	99	96.59
Nucleotide metabolism	pEa_SNUABM32_00235	putative exonuclease	*Dickeya* phage vB_DsoM_AD1	99	98.06

Nucleotide metabolism	pEa_SNUABM32_00245	putative cyclic phosphodiesterase	*Dickeya* phage vB_DsoM_AD1	99	93.55
Nucleotide metabolism	pEa_SNUABM32_00247	putative RpoD subfamily RNA polymerase sigma factor	*Dickeya* phage vB_DsoM_AD1	99	97.39
Nucleotide metabolism	pEa_SNUABM32_00248	putative ssDNA binding protein	*Dickeya* phage vB_DsoM_AD1	63	95.15
Nucleotide metabolism	pEa_SNUABM32_00249	putative replicative DNA helicase	*Dickeya* phage vB_DsoM_AD1	99	87.31
Nucleotide metabolism	pEa_SNUABM32_00271	putative acetyltransferase	*Dickeya* phage vB_DsoM_AD1	99	92.47
Nucleotide metabolism	pEa_SNUABM32_00275	putative RNA 2'-phosphotransferase	*Dickeya* phage vB_DsoM_AD1	97	80.79
Nucleotide metabolism	pEa_SNUABM32_00276	putative radical S-adenosyl-L-methionine protein	*Dickeya* phage vB_DsoM_AD1	99	69.32
Nucleotide metabolism	pEa_SNUABM32_00279	putative DksA/TraR family C4-type zinc finger protein	*Dickeya* phage vB_DsoM_AD1	98	93.1
Nucleotide metabolism	pEa_SNUABM32_00289	putative UV damage repair endonuclease	*Dickeya* phage vB_DsoM_AD1	99	80.98
Nucleotide metabolism	pEa_SNUABM32_00300	putative dUTPase	*Dickeya* phage vB_DsoM_AD1	96	68.11
Lysis	pEa_SNUABM32_00303	putative lytic transglycosylase	*Erwinia* phage vB_EamM_Alexandra	99	88.94
Nucleotide metabolism	pEa_SNUABM32_00305	putative metal-dependent phosphohydrolase	*Dickeya* phage vB_DsoM_AD1	99	60.51
Nucleotide metabolism	pEa_SNUABM32_00330	putative DNA gyrase subunit B	*Dickeya* phage vB_DsoM_AD1	97	91.39
Nucleotide metabolism	pEa_SNUABM32_00331	putative DNA gyrase subunit A	*Erwinia* phage vB_EamM_Y3	97	88.15
Nucleotide metabolism	pEa_SNUABM32_00333	putative endonuclease	*Dickeya* phage vB_DsoM_AD1	99	92.78

Table S5. Functional classification of ORFs in *Erwinia* phage pEa_SNUABM_47.

Group	Locus tag	Encoded protein	Related organism	Query cover (%)	Identiy (%)
Structure & packaging	pEa_SNUABM47_0 0019	putative membrane protein	*Serratia* phage BF	98	100
Additional function	pEa_SNUABM47_0 0021	SPFH domain containing protein	*Serratia* phage BF	99	100
Structure & packaging	pEa_SNUABM47_0 0026	putative structural protein	*Serratia* phage BF	99	99.35
Structure & packaging	pEa_SNUABM47_0 0058	putative membrane protein	*Serratia* phage BF	99	100
Nucleotide metabolism	pEa_SNUABM47_0 0062	putative DNA N-6-adenine-methyltransferase	*Serratia* phage BF	99	100
Nucleotide metabolism	pEa_SNUABM47_0 0063	putative cytitidyltransferase	*Erwinia* phage pEa_SNUABM_12	99	99.44
Nucleotide metabolism	pEa_SNUABM47_0 0071	putative RNA ligase, T4 RnlA family	*Erwinia* phage pEa_SNUABM_12	99	97.74
Nucleotide metabolism	pEa_SNUABM47_0 0075	putative polynucleotide 5'-kinase and 3'-phosphatase	*Erwinia* phage pEa_SNUABM_12	99	100
Structure & packaging	pEa_SNUABM47_0 0079	putative membrane protein	*Serratia* phage BF	98	100
Structure & packaging	pEa_SNUABM47_0 0082	putative membrane protein	*Serratia* phage BF	98	100
Additional function	pEa_SNUABM47_0 0089	putative serine/threonine-protein phosphatase	*Serratia* phage BF	99	100
Nucleotide metabolism	pEa_SNUABM47_0 0092	putative DNA ligase	*Serratia* phage BF	99	99.79
Additional function	pEa_SNUABM47_0 0094	putative serine/threonine-protein phosphatase	*Erwinia* phage pEa_SNUABM_12	99	100
Structure & packaging	pEa_SNUABM47_0 0096	putative ATP-dependent Clp protease proteolytic subunit	*Serratia* phage BF	99	100
Structure & packaging	pEa_SNUABM47_0 0101	putative structural protein	*Serratia* phage BF	99	100
Nucleotide metabolism	pEa_SNUABM47_0 0102	putative CMP/dCMP deaminase	*Erwinia* phage pEa_SNUABM_12	99	99.36
Structure & packaging	pEa_SNUABM47_0 0104	putative major tail protein	*Erwinia* phage pEa_SNUABM_12	99	98.34
Structure & packaging	pEa_SNUABM47_0 0105	putative major tail protein	*Serratia* phage BF	99	100
Nucleotide metabolism	pEa_SNUABM47_0 0107	putative nicotinamide phosphoribosyltransferase	*Erwinia* phage pEa_SNUABM_12	99	100
Structure & packaging	pEa_SNUABM47_0 0108	putative membrane protein	*Serratia* phage BF	98	100
Structure & packaging	pEa_SNUABM47_0 0109	putative structural protein	*Erwinia* phage pEa_SNUABM_12	99	100
Nucleotide metabolism	pEa_SNUABM47_0 0111	putative Sir2-like protein	*Serratia* phage BF	99	100
Nucleotide metabolism	pEa_SNUABM47_0 0112	putative nudix hydrolase	*Serratia* phage BF	99	100
Structure & packaging	pEa_SNUABM47_0 0113	putative membrane protein	*Serratia* phage BF	99	100
Additional function	pEa_SNUABM47_0 0114	putative PhoH family protein	*Serratia* phage BF	99	100
Structure & packaging	pEa_SNUABM47_0 0115	putative prohead core scaffolding protein	*Erwinia* phage pEa_SNUABM_12	99	100
Structure & packaging	pEa_SNUABM47_0 0118	putative membrane protein	*Serratia* phage BF	97	100
Structure & packaging	pEa_SNUABM47_0 0119	putative membrane protein	*Serratia* phage BF	97	100
Structure & packaging	pEa_SNUABM47_0 0123	putative co-chaperonin GroES	*Serratia* phage BF	98	100
tRNA related	pEa_SNUABM47_0 0124	putative tyrosyl-tRNA synthetase	*Serratia* phage BF	99	99.76
Nucleotide metabolism	pEa_SNUABM47_0 0128	putative adenine-specific DNA methylase	*Serratia* phage BF	99	100
Nucleotide metabolism	pEa_SNUABM47_0 0130	putative dCMP deaminase	*Serratia* phage BF	99	100

Nucleotide metabolism	pEa_SNUABM47_0 0131	putative AAA domain-containing ATPase	*Serratia* phage BF	99	99.46
Nucleotide metabolism	pEa_SNUABM47_0 0132	putative anaerobic NTP reductase large subunit	*Serratia* phage BF	99	100
Nucleotide metabolism	pEa_SNUABM47_0 0136	putative pyruvate formate-lyase	*Serratia* phage BF	99	100
Nucleotide metabolism	pEa_SNUABM47_0 0138	putative anaerobic ribonucleoside-triphosphate reductase activating protein	*Erwinia* phage pEa_SNUABM_12	99	100
Structure & packaging	pEa_SNUABM47_0 0140	putative minor tail protein	*Erwinia* phage pEa_SNUABM_12	99	98.89
Structure & packaging	pEa_SNUABM47_0 0141	putative structural protein	*Serratia* phage BF	99	99.89
Structure & packaging	pEa_SNUABM47_0 0142	putative structural protein	*Erwinia* phage pEa_SNUABM_12	99	99.35
tRNA related	pEa_SNUABM47_0 0145	putative tRNAHis-5'-guanylyltransferase	*Erwinia* phage pEa_SNUABM_12	99	100
Nucleotide metabolism	pEa_SNUABM47_0 0147	putative NrdA protein	*Erwinia* phage pEa_SNUABM_12	99	100
Nucleotide metabolism	pEa_SNUABM47_0 0150	putative site-specific DNA methyltransferase	*Serratia* phage BF	99	99.3
Nucleotide metabolism	pEa_SNUABM47_0 0152	putative nucleotidyltransferase	*Serratia* phage BF	99	100
Additional function	pEa_SNUABM47_0 0157	putative acyl carrier protein	*Serratia* phage BF	99	100
Structure & packaging	pEa_SNUABM47_0 0158	putative membrane protein	*Serratia* phage BF	99	99.17
Lysis	pEa_SNUABM47_0 0159	putative o-spannin	*Erwinia* phage pEa_SNUABM_12	99	100
Structure & packaging	pEa_SNUABM47_0 0160	putative structural protein	*Serratia* phage BF	99	100
Nucleotide metabolism	pEa_SNUABM47_0 0163	putative starvation-inducible DNA-binding protein	*Serratia* phage BF	99	100
Nucleotide metabolism	pEa_SNUABM47_0 0167	putative GTP cyclohydrolase	*Serratia* phage BF	99	100
Nucleotide metabolism	pEa_SNUABM47_0 0169	putative thymidine kinase	*Serratia* phage BF	99	99.48
Structure & packaging	pEa_SNUABM47_0 0170	putative tail protein	*Serratia* phage BF	99	100
Structure & packaging	pEa_SNUABM47_0 0171	putative membrane protein	*Serratia* phage BF	99	100
Structure & packaging	pEa_SNUABM47_0 0173	putative membrane protein	*Yersinia* phage fHe-Yen9-03	99	72.73
Nucleotide metabolism	pEa_SNUABM47_0 0175	putative RNA ligase	*Erwinia* phage pEa_SNUABM_12	99	99.76
Structure & packaging	pEa_SNUABM47_0 0179	putative membrane protein	*Serratia* phage BF	97	97.83
Lysis	pEa_SNUABM47_0 0182	putative lysozyme	*Serratia* phage BF	99	99.68
Nucleotide metabolism	pEa_SNUABM47_0 0183	putative sigma 54 modulation protein/ribosomal protein	*Serratia* phage BF	99	100
tRNA related	pEa_SNUABM47_0 0185	putative tRNA nucleotidyl transferase	*Serratia* phage BF	99	100
Nucleotide metabolism	pEa_SNUABM47_0 0186	putative nudix hydrolase	*Serratia* phage BF	99	100
Nucleotide metabolism	pEa_SNUABM47_0 0187	putative glutaredoxin	*Serratia* phage BF	98	100
tRNA related	pEa_SNUABM47_0 0188	putative aspartyl-tRNA amidotransferase	*Serratia* phage BF	99	100
Nucleotide metabolism	pEa_SNUABM47_0 0191	putative Appr-1-p processing enzyme	*Serratia* phage BF	99	99.4
Structure & packaging	pEa_SNUABM47_0 0194	putative neck protein	*Serratia* phage BF	99	100
Nucleotide metabolism	pEa_SNUABM47_0 0196	putative deoxynucleotide monophosphate kinase	*Erwinia* phage pEa_SNUABM_12	99	100
Structure & packaging	pEa_SNUABM47_0 0197	putative tail sheath protein	*Erwinia* phage pEa_SNUABM_12	99	100
Structure & packaging	pEa_SNUABM47_0 0198	putative structural protein	*Erwinia* phage pEa_SNUABM_12	99	100
Structure & packaging	pEa_SNUABM47_0 0199	putative structural protein	*Serratia* phage BF	99	100
Structure & packaging	pEa_SNUABM47_0 0200	putative structural protein	*Serratia* phage BF	99	100

Structure & packaging	pEa_SNUABM47_0 0201	putative head completion protein	*Serratia* phage BF	99	100
Structure & packaging	pEa_SNUABM47_0 0203	putative structural protein	*Erwinia* phage pEa_SNUABM_12	99	100
Structure & packaging	pEa_SNUABM47_0 0204	putative structural protein	*Serratia* phage BF	99	100
Structure & packaging	pEa_SNUABM47_0 0205	putative structural protein	*Serratia* phage BF	99	100
Structure & packaging	pEa_SNUABM47_0 0206	putative structural protein	*Serratia* phage BF	99	99.38
Structure & packaging	pEa_SNUABM47_0 0207	putative structural protein	*Serratia* phage BF	99	100
Nucleotide metabolism	pEa_SNUABM47_0 0209	putative ATPase	*Serratia* phage BF	99	100
Nucleotide metabolism	pEa_SNUABM47_0 0216	putative thymidylate synthase	*Serratia* phage BF	99	100
Structure & packaging	pEa_SNUABM47_0 0217	putative structural protein	*Serratia* phage BF	99	100
Structure & packaging	pEa_SNUABM47_0 0221	putative structural protein	*Serratia* phage BF	99	100
Structure & packaging	pEa_SNUABM47_0 0222	putative long tail fiber proximal subunit	*Erwinia* phage pEa_SNUABM_50	99	97.79
Structure & packaging	pEa_SNUABM47_0 0223	putative structural protein	*Serratia* phage BF	99	100
Structure & packaging	pEa_SNUABM47_0 0224	putative structural protein	*Serratia* phage BF	99	100
Structure & packaging	pEa_SNUABM47_0 0225	putative structural protein	*Serratia* phage BF	99	100
Structure & packaging	pEa_SNUABM47_0 0226	putative structural protein	*Serratia* phage BF	99	100
Structure & packaging	pEa_SNUABM47_0 0227	putative structural protein	*Serratia* phage BF	99	100
Nucleotide metabolism	pEa_SNUABM47_0 0228	putative NUDIX hydrolase family protein	*Serratia* phage BF	99	100
Structure & packaging	pEa_SNUABM47_0 0229	putative tail sheath stabilizer and completion protein	*Serratia* phage BF	99	100
Structure & packaging	pEa_SNUABM47_0 0230	putative structural protein	*Serratia* phage BF	99	100
Structure & packaging	pEa_SNUABM47_0 0231	putative ATP-dependent Clp protease ATP-binding subunit clpA	*Erwinia* phage pEa_SNUABM_12	99	100
Structure & packaging	pEa_SNUABM47_0 0232	putaive structural protein	*Serratia* phage BF	99	99.88
Structure & packaging	pEa_SNUABM47_0 0233	putative baseplate wedge	*Serratia* phage BF	99	99.83
Structure & packaging	pEa_SNUABM47_0 0234	putative baseplate protein	*Serratia* phage BF	99	100
Lysis	pEa_SNUABM47_0 0235	putative baseplate hub subunit and tail lysozyme	*Serratia* phage BF	99	100
Lysis	pEa_SNUABM47_0 0236	putative T4-like phage baseplate hub and tail lysozyme	*Serratia* phage BF	99	100
Structure & packaging	pEa_SNUABM47_0 0237	putative structural protein	*Serratia* phage BF	99	100
Structure & packaging	pEa_SNUABM47_0 0238	putative baseplate wedge protein	*Serratia* phage BF	99	99.04
Structure & packaging	pEa_SNUABM47_0 0239	putative structural protein	*Serratia* phage BF	99	100
Nucleotide metabolism	pEa_SNUABM47_0 0240	putative RNA sigma factor for late transcription	*Serratia* phage BF	99	100
Nucleotide metabolism	pEa_SNUABM47_0 0241	putative endonuclease subunit	*Serratia* phage BF	99	100
Nucleotide metabolism	pEa_SNUABM47_0 0242	putative endonuclease subunit	*Erwinia* phage pEa_SNUABM_12	99	99.86
Structure & packaging	pEa_SNUABM47_0 0243	putative EndoVII packaging and recombination endonuclease	*Serratia* phage BF	99	100
Structure & packaging	pEa_SNUABM47_0 0245	putative baseplate hub subunit	*Serratia* phage BF	99	100
Structure & packaging	pEa_SNUABM47_0 0246	putative tape measure protein	*Serratia* phage BF	99	99.86
Structure & packaging	pEa_SNUABM47_0 0247	putative portal vertex protein	*Serratia* phage BF	99	100
Structure & packaging	pEa_SNUABM47_0 0249	putative structural protein	*Serratia* phage BF	99	99.66

Structure & packaging	pEa_SNUABM47_00250	putative prohead core protein	*Serratia* phage BF	99	100
Structure & packaging	pEa_SNUABM47_00251	putative scaffolding protein	*Serratia* phage BF	99	100
Structure & packaging	pEa_SNUABM47_00252	putative major capsid protein	*Serratia* phage BF	99	100
Nucleotide metabolism	pEa_SNUABM47_00254	putative GIY-YIG nuclease family protein	*Serratia* phage BF	99	100
Structure & packaging	pEa_SNUABM47_00255	putative tail fiber protein	*Serratia* phage BF	99	99.82
Nucleotide metabolism	pEa_SNUABM47_00258	putative DNA polymerase	*Serratia* phage BF	99	99.9
Additional function	pEa_SNUABM47_00261	putative serine/threonine protein phosphatase	*Serratia* phage BF	99	100
Additional function	pEa_SNUABM47_00262	putative type I antifreeze protein	*Serratia* phage BF	98	100
Structure & packaging	pEa_SNUABM47_00263	putative co-chaperonin GroES	*Serratia* phage BF	98	100
Structure & packaging	pEa_SNUABM47_00264	putative structural protein	*Serratia* phage BF	99	100
Structure & packaging	pEa_SNUABM47_00265	putative structural protein	*Serratia* phage BF	99	100
Structure & packaging	pEa_SNUABM47_00266	putative structural protein	*Serratia* phage BF	99	100
Nucleotide metabolism	pEa_SNUABM47_00267	putative RNaseH ribonuclease	*Serratia* phage BF	99	100
Structure & packaging	pEa_SNUABM47_00269	putative terminase like protein	*Erwinia* phage pEa_SNUABM_12	99	100
Structure & packaging	pEa_SNUABM47_00270	putative terminase large subunit	*Serratia* phage BF	99	100
Structure & packaging	pEa_SNUABM47_00271	putative structural protein	*Erwinia* phage pEa_SNUABM_12	99	100
Nucleotide metabolism	pEa_SNUABM47_00272	putative ssDNA binding protein	*Serratia* phage BF	99	100
Nucleotide metabolism	pEa_SNUABM47_00273	putative UvsX protein	*Serratia* phage BF	99	100
Nucleotide metabolism	pEa_SNUABM47_00274	putative UvsY portein	*Serratia* phage BF	99	100
Nucleotide metabolism	pEa_SNUABM47_00275	putative DNA polymerase III epsilon subunit	*Serratia* phage BF	99	100
Nucleotide metabolism	pEa_SNUABM47_00276	putative RNA-DNA + DNA-DNA helicase	*Serratia* phage BF	99	100
Structure & packaging	pEa_SNUABM47_00281	putative structural protein	*Serratia* phage BF	99	100
Structure & packaging	pEa_SNUABM47_00282	putative membrane protein	*Serratia* phage BF	99	100
Structure & packaging	pEa_SNUABM47_00284	putative structural protein	*Serratia* phage BF	99	100
Nucleotide metabolism	pEa_SNUABM47_00285	putative DNA primase subunit	*Serratia* phage BF	99	99.71
Nucleotide metabolism	pEa_SNUABM47_00286	putative DNA primase-helicase	*Serratia* phage BF	99	100
Structure & packaging	pEa_SNUABM47_00290	putative structural protein	*Erwinia* phage pEa_SNUABM_12	99	100
Structure & packaging	pEa_SNUABM47_00291	putative structural protein	*Serratia* phage BF	98	100
Structure & packaging	pEa_SNUABM47_00293	putative structural protein	*Erwinia* phage pEa_SNUABM_12	99	100
Structure & packaging	pEa_SNUABM47_00294	putative structural protein	*Serratia* phage BF	99	100
Nucleotide metabolism	pEa_SNUABM47_00295	putative restriction endonuclease type II like-protein	*Serratia* phage BF	99	100
Structure & packaging	pEa_SNUABM47_00297	putative structural protein	*Serratia* phage BF	99	100
Nucleotide metabolism	pEa_SNUABM47_00298	putative aerobic ribonucleotide-diphosphate reductase alpha subunit	*Serratia* phage BF	99	100
Nucleotide metabolism	pEa_SNUABM47_00299	putative aerobic ribonucleotide-diphosphate reductase beta subunit	*Serratia* phage BF	99	100
Structure & packaging	pEa_SNUABM47_00300	putative membrane protein	*Serratia* phage BF	98	100
Structure & packaging	pEa_SNUABM47_00302	putative structural protein	*Serratia* phage BF	99	100

Nucleotide metabolism	pEa_SNUABM47_0 0303	putative nucleotide pyrophosphohydrolase	*Erwinia* phage pEa_SNUABM_12	99	100
Structure & packaging	pEa_SNUABM47_0 0305	putative structural protein	*Serratia* phage BF	99	100
Structure & packaging	pEa_SNUABM47_0 0306	putative membrane protein	*Serratia* phage BF	99	100
Nucleotide metabolism	pEa_SNUABM47_0 0307	putative dihydrofolate reductase	*Serratia* phage BF	99	98.28
Nucleotide metabolism	pEa_SNUABM47_0 0308	putative ribonuclease H	*Serratia* phage BF	99	100
Nucleotide metabolism	pEa_SNUABM47_0 0309	putative DNA helicase Dda	*Serratia* phage BF	99	100
Structure & packaging	pEa_SNUABM47_0 0311	putative structural protein	*Erwinia* phage pEa_SNUABM_12	99	100
Nucleotide metabolism	pEa_SNUABM47_0 0313	putative translation initiation factor IF-3	*Serratia* phage BF	99	100
Structure & packaging	pEa_SNUABM47_0 0314	putative ATP-dependent Clp protease	*Serratia* phage BF	99	100
Nucleotide metabolism	pEa_SNUABM47_0 0315	putative DnaJ-like protein	*Serratia* phage BF	99	100
Structure & packaging	pEa_SNUABM47_0 0316	putative structural protein	*Serratia* phage BF	99	99.81
Structure & packaging	pEa_SNUABM47_0 0317	putative membrane protein	*Serratia* phage BF	99	99.75
Nucleotide metabolism	pEa_SNUABM47_0 0319	putative topoisomerase II large subunit	*Serratia* phage BF	99	100
Nucleotide metabolism	pEa_SNUABM47_0 0320	putative DNA topoisomerase II medium subunit	*Serratia* phage BF	99	100
Structure & packaging	pEa_SNUABM47_0 0321	putative structural protein	*Serratia* phage BF	99	99.9
Nucleotide metabolism	pEa_SNUABM47_0 0323	putative DNA polymerase III alpha subunit	*Serratia* phage BF	99	100
Structure & packaging	pEa_SNUABM47_0 0325	putative co-chaperonin GroES	*Serratia* phage BF	99	100
Nucleotide metabolism	pEa_SNUABM47_0 0327	putative sliding clamp loader subunit	*Serratia* phage BF	99	100
Additional function	pEa_SNUABM47_0 0328	putative phosphoglycolate phosphatase	*Serratia* phage BF	99	100
Structure & packaging	pEa_SNUABM47_0 0329	putaive structural protein	*Serratia* phage BF	99	100
Structure & packaging	pEa_SNUABM47_0 0330	putaive structural protein	*Serratia* phage BF	99	100
Additional function	pEa_SNUABM47_0 0335	putative TelA like protein	*Serratia* phage BF	99	99.74
Nucleotide metabolism	pEa_SNUABM47_0 0336	putative nucleotide reductase subunit C	*Serratia* phage BF	99	100
Additional function	pEa_SNUABM47_0 0337	putative metallopeptidase	*Serratia* phage BF	99	100
Structure & packaging	pEa_SNUABM47_0 0338	putative membrane protein	*Serratia* phage BF	99	100
Structure & packaging	pEa_SNUABM47_0 0339	putative structural protein	*Serratia* phage BF	99	100
Structure & packaging	pEa_SNUABM47_0 0343	putative membrane protein	*Serratia* phage BF	99	100
Nucleotide metabolism	pEa_SNUABM47_0 0356	putative nicotinamide nucleotide adenylyltransferase	*Serratia* phage BF	99	99.72
Nucleotide metabolism	pEa_SNUABM47_0 0357	putative nicotinamide mononucleotide transporter PnuC	*Serratia* phage BF	99	100
Structure & packaging	pEa_SNUABM47_0 0364	putative structural protein	*Serratia* phage BF	99	100
tRNA	pEa_SNUABM47_0 0368	tRNA-Ser			
Nucleotide metabolism	pEa_SNUABM47_0 0373	putative ATPase	*Serratia* phage BF	99	100
tRNA	pEa_SNUABM47_0 0374	tRNA-Trp			
Nucleotide metabolism	pEa_SNUABM47_0 0376	putative nucleotidase	*Serratia* phage BF	99	100
tRNA	pEa_SNUABM47_0 0379	tRNA-Thr			
tRNA	pEa_SNUABM47_0 0381	tRNA-Leu			

tRNA related	pEa_SNUABM47_0 0392	putative peptidyl-tRNA hydrolase	*Serratia* phage BF	99	100
tRNA	pEa_SNUABM47_0 0393	tRNA-Leu			
Structure & packaging	pEa_SNUABM47_0 0394	putative membrane protein	*Serratia* phage BF	99	100
tRNA	pEa_SNUABM47_0 0412	tRNA-Arg			
tRNA	pEa_SNUABM47_0 0418	tRNA-Pyl			
tRNA	pEa_SNUABM47_0 0420	tRNA-Met			
tRNA	pEa_SNUABM47_0 0426	tRNA-Leu			
tRNA	pEa_SNUABM47_0 0439	tRNA-Phe			
tRNA	pEa_SNUABM47_0 0444	tRNA-Lys			
Structure & packaging	pEa_SNUABM47_0 0448	putative membrane protein	*Serratia* phage BF	99	99.12
tRNA	pEa_SNUABM47_0 0449	tRNA-Leu			
Structure & packaging	pEa_SNUABM47_0 0452	putative membrane protein	*Serratia* phage BF	99	100
Nucleotide metabolism	pEa_SNUABM47_0 0454	putative AAA family ATPase	*Serratia* phage BF	99	100
tRNA	pEa_SNUABM47_0 0456	tRNA-Glu			
tRNA	pEa_SNUABM47_0 0457	tRNA-Ser			
tRNA	pEa_SNUABM47_0 0459	tRNA-Ser			
tRNA	pEa_SNUABM47_0 0460	tRNA-Ser			
Structure & packaging	pEa_SNUABM47_0 0462	putative ATP-dependent Clp protease proteolytic subunit	*Serratia* phage BF	99	100
tRNA	pEa_SNUABM47_0 0465	tRNA-Ile			
tRNA	pEa_SNUABM47_0 0469	tRNA-Asn			
tRNA	pEa_SNUABM47_0 0470	tRNA-Gln			
tRNA	pEa_SNUABM47_0 0471	tRNA-Gly			
tRNA	pEa_SNUABM47_0 0476	tRNA-Asp			
tRNA	pEa_SNUABM47_0 0478	tRNA-Arg			
tRNA	pEa_SNUABM47_0 0483	tRNA-Pro			
tRNA	pEa_SNUABM47_0 0484	tRNA-Pro			
tRNA	pEa_SNUABM47_0 0485	tRNA-Pro			
tRNA	pEa_SNUABM47_0 0486	tRNA-Val			
tRNA	pEa_SNUABM47_0 0489	tRNA-His			
tRNA	pEa_SNUABM47_0 0490	tRNA-Phe			
tRNA	pEa_SNUABM47_0 0492	tRNA-Lys			
Structure & packaging	pEa_SNUABM47_0 0496	putative membrane protein	*Erwinia* phage pEa_SNUABM_12	99	100
Structure & packaging	pEa_SNUABM47_0 0497	putative membrane protein	*Serratia* phage BF	99	99.03
tRNA	pEa_SNUABM47_0 0500	tRNA-Tyr			
tRNA	pEa_SNUABM47_0 0505	tRNA-Cys			
tRNA	pEa_SNUABM47_0 0506	tRNA-Lys			

tRNA	pEa_SNUABM47_0 0508	tRNA-Met			
tRNA	pEa_SNUABM47_0 0509	tRNA-Met			
tRNA	pEa_SNUABM47_0 0510	tRNA-Ala			
Structure & packaging	pEa_SNUABM47_0 0511	putative structural protein	*Erwinia* phage pEa_SNUABM_12	99	100
Nucleotide metabolism	pEa_SNUABM47_0 0512	putative S-adenosyl-L- methionine-dependent methyltransferase	*Serratia* phage BF	99	100
Structure & packaging	pEa_SNUABM47_0 0513	putative structural protein	*Erwinia* phage pEa_SNUABM_12	99	99.73
Structure & packaging	pEa_SNUABM47_0 0519	putative membrane protein	*Serratia* phage BF	99	100
Structure & packaging	pEa_SNUABM47_0 0520	putative membrane protein	*Erwinia* phage pEa_SNUABM_12	99	100
Nucleotide metabolism	pEa_SNUABM47_0 0532	putative subfamily RNA polymerase sigma-70 subunit	*Serratia* phage BF	99	99.7
Structure & packaging	pEa_SNUABM47_0 0535	putative structural protein	*Serratia* phage BF	98	100
Structure & packaging	pEa_SNUABM47_0 0536	putative membrane protein	*Erwinia* phage pEa_SNUABM_12	98	100
Additional function	pEa_SNUABM47_0 0540	putative C4-type zinc finger domain-containing protein	*Serratia* phage BF	98	98.84
Structure & packaging	pEa_SNUABM47_0 0548	putative HNH endonuclease	*Serratia* phage BF	99	100
Structure & packaging	pEa_SNUABM47_0 0554	putative HNH endonuclease	*Erwinia* phage pEa_SNUABM_12	99	100
Nucleotide metabolism	pEa_SNUABM47_0 0555	putative thioredoxin	*Erwinia* phage pEa_SNUABM_12	99	100
Structure & packaging	pEa_SNUABM47_0 0556	putative membrane protein	*Serratia* phage BF	99	100
Structure & packaging	pEa_SNUABM47_0 0558	putative membrane protein	*Serratia* phage BF	99	100
Structure & packaging	pEa_SNUABM47_0 0559	putative membrane protein	*Erwinia* phage pEa_SNUABM_12	99	98.33
Structure & packaging	pEa_SNUABM47_0 0560	putative membrane protein	*Serratia* phage BF	99	100
Structure & packaging	pEa_SNUABM47_0 0561	putative membrane protein	*Erwinia* phage pEa_SNUABM_12	99	99.09
Structure & packaging	pEa_SNUABM47_0 0562	putative membrane protein	*Serratia* phage BF	98	100
Structure & packaging	pEa_SNUABM47_0 0563	putative membrane protein	*Serratia* phage BF	99	100
Additional function	pEa_SNUABM47_0 0566	putative PE-PGRS family protein	*Erwinia* phage pEa_SNUABM_12	99	99.29
Nucleotide metabolism	pEa_SNUABM47_0 0567	putative DNA condensation protein	*Erwinia* phage pEa_SNUABM_12	99	99.47
Nucleotide metabolism	pEa_SNUABM47_0 0571	putative DNA condensation protein	*Erwinia* phage pEa_SNUABM_50	99	97.92
Nucleotide metabolism	pEa_SNUABM47_0 0572	putative DNA condensation protein	*Erwinia* phage pEa_SNUABM_12	99	98.44
Structure & packaging	pEa_SNUABM47_0 0574	putative structural protein	*Serratia* phage BF	99	100
Nucleotide metabolism	pEa_SNUABM47_0 0575	putative DNA condensation protein	*Erwinia* phage pEa_SNUABM_12	99	100

Table S6. Functional classification of ORFs in *Erwinia* phage pEa_SNUABM_48.

Group	Locus tag	Encoded protein	Related organism	Query cover (%)	Identity (%)
Nucleotide metabolism	pEa_SNUABM48_00002	putative RNA polymerase beta subunit	*Salmonella* phage vB_SalM_SA002	99	91.09
Structure & packaging	pEa_SNUABM48_00007	putative portal protein	*Salmonella* phage vB_SalM_SA002	88	72.73
Structure & packaging	pEa_SNUABM48_00008	putative virion structural protein	*Salmonella* phage vB_SalM_SA002	99	78.29
Structure & packaging	pEa_SNUABM48_00009	putative virion structural protein	*Salmonella* phage vB_SalM_SA002	99	90.14
Structure & packaging	pEa_SNUABM48_00010	putative virion structural protein	*Salmonella* phage vB_SalM_SA002	99	86.13
Structure & packaging	pEa_SNUABM48_00011	putative virion structural protein	*Salmonella* phage vB_SalM_SA002	99	90.17
Nucleotide metabolism	pEa_SNUABM48_00013	putative RuvC holliday junction resolvasome	*Salmonella* phage vB_SalM_SA002	99	82.35
Structure & packaging	pEa_SNUABM48_00017	putative virion structural protein	*Salmonella* phage vB_SalM_SA002	99	88.51
Structure & packaging	pEa_SNUABM48_00018	putative virion structural protein	*Salmonella* phage vB_SalM_SA002	99	79.26
Structure & packaging	pEa_SNUABM48_00019	putative membrane protein	*Salmonella* phage vB_SalM_SA002	99	78.55
Structure & packaging	pEa_SNUABM48_00020	putative virion structural protein	*Salmonella* phage vB_SalM_SA002	99	65.33
Structure & packaging	pEa_SNUABM48_00021	putative membrane protein	*Salmonella* phage vB_SalM_SA002	99	78.78
Structure & packaging	pEa_SNUABM48_00022	putative baseplate spike protein	*Salmonella* phage vB_SalM_SA002	94	82.39
Nucleotide metabolism	pEa_SNUABM48_00024	putative SbcC-like nuclease	*Salmonella* phage vB_SalM_SA002	99	78.05
Lysis	pEa_SNUABM48_00031	putative lytic transglycosylase	*Salmonella* phage vB_SalM_SA002	99	84.84
Additional function	pEa_SNUABM48_00032	putative translation regulator	*Salmonella* phage vB_SalM_SA002	99	57.05
Nucleotide metabolism	pEa_SNUABM48_00037	putative 5'(3')-deoxyribonucleotidase	*Salmonella* phage vB_SalM_SA002	93	47.45
Structure & packaging	pEa_SNUABM48_00043	putative structural protein (LemA domain containing)	*Salmonella* phage vB_SalM_SA002	99	81.28
Nucleotide metabolism	pEa_SNUABM48_00046	putative transcription regulator	*Salmonella* phage vB_SalM_SA002	94	82.69
Nucleotide metabolism	pEa_SNUABM48_00048	putative exoribonuclease	*Salmonella* phage vB_SalM_SA002	98	77.21
Nucleotide metabolism	pEa_SNUABM48_00053	putative exodeoxyribonuclease	*Salmonella* phage vB_SalM_SA002	99	73.64
Additional function	pEa_SNUABM48_00056	putative thymidylate synthase	*Salmonella* phage vB_SalM_SA002	99	77.81
Additional function	pEa_SNUABM48_00059	putative nicotinamide mononucleotide transporter	*Salmonella* phage vB_SalM_SA002	99	73.33
Nucleotide metabolism	pEa_SNUABM48_00066	putative phosphohydrolase	*Erwinia* phage vB EamM RAY	85	40
Nucleotide metabolism	pEa_SNUABM48_00076	putative thymidylate kinase	*Salmonella* phage vB_SalM_SA002	54	37.38
Nucleotide metabolism	pEa_SNUABM48_00077	putative NAD-dependent DNA ligase	*Erwinia* typographi	99	46.33
Nucleotide metabolism	pEa_SNUABM48_00081	putative nucleoside triphosphate pyrophosphohydrolase	*Salmonella* phage vB_SalM_SA002	82	55.58
Additional function	pEa_SNUABM48_00086	putative YgiB domain containing protein	*Salmonella* phage vB_SalM_SA002	99	86.05
Structure & packaging	pEa_SNUABM48_00092	putative tail protein (DUF1353 domain containing)	*Salmonella* phage vB_SalM_SA002	99	78.45
Structure & packaging	pEa_SNUABM48_00104	putative virion structural protein (collagen like)	*Serratia* phage PCH45	57	31.69
Structure & packaging	pEa_SNUABM48_00105	putative virion structural protein (collagen like)	*Serratia* phage PCH45	49	37.50
Structure & packaging	pEa_SNUABM48_00106	putative virion structural protein	*Salmonella* phage vB_SalM_SA002	91	41.75

Structure & packaging	pEa_SNUABM48_00107	putative virion structuralprotein	*Salmonella* phage vB_SalM_SA002	99	42.14
Structure & packaging	pEa_SNUABM48_00108	putative virion structural protein	*Salmonella* phage vB_SalM_SA002	99	41.9
Structure & packaging	pEa_SNUABM48_00109	putative virion structural protein	*Salmonella* phage vB_SalM_SA002	99	45.86
Structure & packaging	pEa_SNUABM48_00110	putative virion structural protein	*Salmonella* phage vB_SalM_SA002	99	66.19
Structure & packaging	pEa_SNUABM48_00111	putative virion structural protein	*Salmonella* phage vB_SalM_SA002	99	73.21
Nucleotide metabolism	pEa_SNUABM48_00115	putative RNA ligase	*Proteus* phage 10	99	53.72
Nucleotide metabolism	pEa_SNUABM48_00116	putative tRNA splicing ligase	*Erwinia* phage vB EamM RAY	99	83.74
Nucleotide metabolism	pEa_SNUABM48_00117	putative thymidine kinase	*Salmonella* phage vB_SalM_SA002	98	67.35
Nucleotide metabolism	pEa_SNUABM48_00124	putative NAD-dependent DNA ligase	*Salmonella* phage vB_SalM_SA002	99	46.8
Nucleotide metabolism	pEa_SNUABM48_00125	putative dihydrofolate reductase	*Salmonella* phage vB_SalM_SA002	99	47.46
Structure & packaging	pEa_SNUABM48_00129	putative virion structural protein	*Erwinia* phage vB EamM RisingSun	76	36.44
Structure & packaging	pEa_SNUABM48_00130	putative virion structural protein	*Erwinia* phage vB EamM RisingSun	85	35.71
Structure & packaging	pEa_SNUABM48_00131	putative virion structural protein	*Erwinia* phage vB EamM RisingSun	99	35.64
Nucleotide metabolism	pEa_SNUABM48_00133	putative DEAD-like helicase	*Salmonella* phage vB_SalM_SA002	99	82.29
Structure & packaging	pEa_SNUABM48_00149	putative virion structural protein	*Salmonella* phage vB_SalM_SA002	99	82.65
Nucleotide metabolism	pEa_SNUABM48_00151	putative ribonuclease HI	*Salmonella* phage vB_SalM_SA002	99	87.5
Nucleotide metabolism	pEa_SNUABM48_00154	putative UvsX protein	*Salmonella* phage vB_SalM_SA002	99	89.83
Nucleotide metabolism	pEa_SNUABM48_00155	putative Rossmann fold nucleotide binding protein	*Salmonella* phage vB_SalM_SA002	99	80.43
Nucleotide metabolism	pEa_SNUABM48_00156	putative Rossmann fold nucleotide binding protein	*Salmonella* phage vB_SalM_SA002	75	81.04
Structure & packaging	pEa_SNUABM48_00157	putative virion associated RNA polymerase	*Salmonella* phage vB_SalM_SA002	99	86.94
Lysis	pEa_SNUABM48_00168	putative structural peptidoglycan hydrolase	*Salmonella* phage vB_SalM_SA002	99	75.23
Nucleotide metabolism	pEa_SNUABM48_00169	putative virion associated RNA polymerase	*Salmonella* phage vB_SalM_SA002	99	87.73
Nucleotide metabolism	pEa_SNUABM48_00170	putative virion associated RNA polymerase	*Salmonella* phage vB_SalM_SA002	99	84.34
Lysis	pEa_SNUABM48_00171	putative lytic transglycosylase	*Salmonella* phage vB_SalM_SA002	99	87.5
Structure & packaging	pEa_SNUABM48_00176	putative prohead core protease	*Salmonella* phage vB_SalM_SA002	99	89.23
Structure & packaging	pEa_SNUABM48_00188	putative tail tube protein	*Salmonella* phage vB_SalM_SA002	98	95.45
Structure & packaging	pEa_SNUABM48_00189	putative tail sheath protein	*Salmonella* phage vB_SalM_SA002	99	83
Structure & packaging	pEa_SNUABM48_00190	putative virion structural protein	*Salmonella* phage vB_SalM_SA002	99	77.85
Structure & packaging	pEa_SNUABM48_00191	putative virion structural protein	*Salmonella* phage vB_SalM_SA002	99	84.11
Structure & packaging	pEa_SNUABM48_00192	putative virion structural protein	*Salmonella* phage vB_SalM_SA002	99	81.71
Structure & packaging	pEa_SNUABM48_00193	putative terminase large subunit	*Salmonella* phage vB_SalM_SA002	99	92.42
Structure & packaging	pEa_SNUABM48_00211	putative phiKZ like phage internal head protein	*Salmonella* phage vB_SalM_SA002	94	58.92
tRNA	pEa_SNUABM48_00224	tRNA			
Nucleotide metabolism	pEa_SNUABM48_00225	putative methionine-tRNA ligase	*Klebsiella* phage N1M2	88	55.26
tRNA	pEa_SNUABM48_00228	tRNA			
Nucleotide metabolism	pEa_SNUABM48_00233	puptative RNA polymerase	*Salmonella* phage vB_SalM_SA002	99	68.37
Nucleotide metabolism	pEa_SNUABM48_00236	putative NAD-dependent DNA ligase	*Salmonella* phage vB_SalM_SA002	79	44.3

Structure & packaging	pEa_SNUABM48_00241	putative phage tubulin like protein	*Salmonella* phage vB_SalM_SA002	99	87.73
Nucleotide metabolism	pEa_SNUABM48_00251	putative DNA-directed DNA polymerase	*Salmonella* phage vB_SalM_SA002	99	72.73
Nucleotide metabolism	pEa_SNUABM48_00253	putative phiKZ gp105-like protein	*Salmonella* phage vB_SalM_SA002	99	74.13
Nucleotide metabolism	pEa_SNUABM48_00254	putative DNA-directed RNA polymerase betasubunit	*Salmonella* phage vB_SalM_SA002	99	87.14
Nucleotide metabolism	pEa_SNUABM48_00274	putative exonuclease	*Salmonella* phage vB_SalM_SA002	99	82.79
Structure & packaging	pEa_SNUABM48_00277	putative decoration protein	*Salmonella* phage vB_SalM_SA002	98	76.4
Nucleotide metabolism	pEa_SNUABM48_00282	putative DNA-directed RNA polymerase beta subunit	*Salmonella* phage vB_SalM_SA002	99	89.2
Nucleotide metabolism	pEa_SNUABM48_00283	putative DNA-directed RNA polymerase beta subunit	*Salmonella* phage vB_SalM_SA002	99	90.38
Nucleotide metabolism	pEa_SNUABM48_00284	putative ATP dependent DNA helicase	*Erwinia* phage vB EamM RAY	99	65.38
Structure & packaging	pEa_SNUABM48_00307	putative baseplate hub assembly protein	*Salmonella* phage vB_SalM_SA002	99	65.35
Nucleotide metabolism	pEa_SNUABM48_00310	putative DNA-directed RNA polymerase beta subunit	*Salmonella* phage vB_SalM_SA002	99	79.13
Nucleotide metabolism	pEa_SNUABM48_00314	putative (p)ppGpp synthase/hydrolase HD domain			
Nucleotide metabolism	pEa_SNUABM48_00324	putative DNA polymerase	*Salmonella* phage vB_SalM_SA002	99	91.67
Structure & packaging	pEa_SNUABM48_00325	putative virion structural protein	*Salmonella* phage vB_SalM_SA002	99	73.62
Structure & packaging	pEa_SNUABM48_00329	putative virion structural protein	*Salmonella* phage vB_SalM_SA002	99	79.37
Structure & packaging	pEa_SNUABM48_00330	putative virion structural protein	*Salmonella* phage vB_SalM_SA002	99	86.54
Structure & packaging	pEa_SNUABM48_00334	putative phiKZ like phage internal head protein	*Salmonella* phage vB_SalM_SA002	99	65.79
Structure & packaging	pEa_SNUABM48_00337	putative phiKZ like phage internal head protein	*Salmonella* phage vB_SalM_SA002	82	60.24
Structure & packaging	pEa_SNUABM48_00343	putative virion structural protein (collagen like)	*Salmonella* phage vB_SalM_SA002	99	53.39
Structure & packaging	pEa_SNUABM48_00344	putative virion structural protein	*Salmonella* phage vB_SalM_SA002	99	83.24
Structure & packaging	pEa_SNUABM48_00346	putative virion structural protein	*Salmonella* phage vB_SalM_SA002	99	70.53
Structure & packaging	pEa_SNUABM48_00348	putative virion structural protein	*Salmonella* phage vB_SalM_SA002	99	81.92
Nucleotide metabolism	pEa_SNUABM48_00351	putative thymidylate kinase	*Salmonella* phage vB_SalM_SA002	97	64.93
Nucleotide metabolism	pEa_SNUABM48_00356	putative helicase	*Salmonella* phage vB_SalM_SA002	99	93.14
Structure & packaging	pEa_SNUABM48_00358	putative major capsid protein	*Salmonella* phage vB_SalM_SA002	99	86.91
